# DNA Authentication of St John’s Wort (*Hypericum perforatum* L.) Commercial Products Targeting the ITS Region

**DOI:** 10.3390/genes10040286

**Published:** 2019-04-09

**Authors:** Caroline Howard, Eleanor Hill, Marco Kreuzer, Purvi Mali, Eva Masiero, Adrian Slater, Tiziana Sgamma

**Affiliations:** 1 Biomolecular Technology Group, Leicester School of Allied Health Science, Faculty of Health and Life Sciences, De Montfort University, Leicester LE1 9BH, UK; eleanorhill.0@gmail.com (E.H.); p11042664@my365.dmu.ac.uk (P.M.); eva.masiero@dmu.ac.uk (E.M.); 2 National Institute for Biological Standards and Control, Blanche Lane, South Mimms, Potters Bar, Hertfordshire EN6 3QG, UK; marcokrz@gmail.com

**Keywords:** *Hypericum perforatum*, St John’s Wort, barcoding, metabarcoding, DNA fragmentation, medicinal plant extract, qPCR

## Abstract

There is considerable potential for the use of DNA barcoding methods to authenticate raw medicinal plant materials, but their application to testing commercial products has been controversial. A simple PCR test targeting species-specific sequences within the nuclear ribosomal internal transcribed spacer (ITS) region was adapted to screen commercial products for the presence of *Hypericum perforatum* L. material. DNA differing widely in amount and extent of fragmentation was detected in a number of product types. Two assays were designed to further analyse this DNA using a curated database of selected *Hypericum* ITS sequences: A qPCR assay based on a species-specific primer pair spanning the ITS1 and ITS2 regions, using synthetic DNA reference standards for DNA quantitation and a Next Generation Sequencing (NGS) assay separately targeting the ITS1 and ITS2 regions. The ability of the assays to detect *H. perforatum* DNA sequences in processed medicines was investigated. Out of twenty different matrices tested, both assays detected *H. perforatum* DNA in five samples with more than 10^3^ ITS copies µL^−1^ DNA extract, whilst the qPCR assay was also able to detect lower levels of DNA in two further samples. The NGS assay confirmed that *H. perforatum* was the major species in all five positive samples, though trace contaminants were also detected.

## 1. Introduction

Traditional plant-based medicines were once used as the primary source of healthcare, but since the advent of modern pharmaceuticals these preparations have been relegated to the status of ‘complementary’ medicine. In more developed countries, and particularly in the UK, the use of ‘herbal medicines’ has dramatically reduced over the past century. However, recent years have seen the resurgence of biologically active botanicals in many different guises: Food supplements, herbal medicines, nutraceuticals, natural health products and herbal remedies. Each of these types of product have been gaining in popularity, with a recent report showing that the sales of dietary supplements in the US are worth more than ever [1]. Each different form of product has different requirements in terms of regulation [2], although it is understood that end users are not necessarily aware of what type of product they are purchasing, and much less what effect that infers on the quality of the product. The booming market for botanicals has been felt in the areas of production; few of the herbals on the market are cultivated and the majority are wild harvested. This has had a drastic effect on the natural populations of some plants, with many medicinal plants listed by the Convention on International Trade in Endangered Species of Wild Fauna and Flora (CITES) [3,4] and the number increasing year by year [5]. The ‘perfect storm’ of high demand, low availability and little regulation is the perfect environment for adulteration and contamination of the supply chain [4,6].

As one of the leading traditional herbal medicines in the world, St John’s Wort (*Hypericum perforatum* L.) has been the focus of many different techniques for the assessment of quality, efficacy and safety [7,8,9,10,11,12,13,14,15,16]. This position in the market place also attracted the application of novel, DNA-based, identification techniques as long ago as 2004 [11]. The identification of plant material using full DNA barcodes has become reasonably standard in research terms, and has even been published as a method within the British Pharmacopoeia for routine use in industry [17]. However, there are many distinct limitations to the use of full barcode regions for identity:

1. Time, expense and expertise required for DNA sequencing and analysis.
2. Inability to detect adulterant/contaminant material.
3. Limited use with degraded DNA.

Work in this research group initially developed a quick and easy PCR test for the presence of *H. perforatum* DNA and showed that this worked well in a small selection of finished products [13]. The manufacturing process for herbal over-the-counter (OTC) products can damage the DNA of the ‘target’ plant material, meaning that it can become degraded. This was indeed empirically shown by the size of amplifiable DNA products within each extraction; as the amplicon length decreased so the chance of amplifying a product increased. The importance of selecting short ‘mini-codes’ from within larger barcode regions to circumvent this DNA fragmentation was recognised, and the group developed a test resulting in an 80 bp PCR product. This was successfully applied to a sample set consisting of capsules, tables and tinctures [18]. 

The development of identification methods in all scientific disciplines is, naturally, a product of the current ‘state-of-the-art’, and as such requires revision over time as methods and technologies develop, and as further information becomes available due to scientific endeavour. Thus, the importance of following an iterative process for test development is imperative, as the authors have previously argued [19]. The increase in the number of sequences available in public databases for *Hypericum* species in the last 10 years has been dramatic, and this extra information needs to be taken into account and incorporated into method maintenance. This has been applied to the assays discussed in this work, redeveloping and applying further checks now that more information is available. The result is an improved technique informed by the current knowledge base and with the added benefit of being a quantitative PCR (qPCR) assay. This development means that the number of starting DNA molecules can be calculated, to a degree. The advantages of using qPCR over barcode sequencing methodologies are numerous, and of particular relevance to industry:

1. Application to degraded samples due to the dramatically reduced amplicon size.
2. No requirement to sequence the amplicons.
3. No requirement to analyse sequencing data.
4. All results from one machine, and one operator.

While qPCR methods present many advantages over conventional PCR and Sanger sequencing of full barcode regions, it must also be noted that the problem of mixed, adulterated or contaminated samples is not sufficiently covered by a single assay. In this paper we propose the pairing of a species-specific qPCR test with a generic qPCR assay for all amplifiable plant DNA. By providing a measure of ‘total DNA’ and ‘specific DNA’ in a sample, a measure of purity is possible [20]. However, the robustness and validity of this technique must be proven, and for this purpose we also analyse the sample set using an alternate technique, which is itself a contender for the most powerful method by which to answer the question ‘what is in a mixed plant sample?’: Next Generation Sequencing (NGS).

Since the development of the original PCR test for *H. perforatum*, the technology available has changed dramatically. The advent of NGS technologies promises to revolutionise many fields, and the identification of botanicals is no exception. The benefits of this sequencing method over Sanger sequencing are profound, as millions of template molecules are individually sequenced at the same time in a ‘massively parallel’ process. This means that mixed samples can be recognised and the different contributors identified if they have previously been sequenced, and that the detection levels are much lower, giving superior sensitivity. The most well published application of NGS methodologies to botanicals is ‘meta-barcoding’, a method in which ‘barcode’ regions are amplified and all the resulting amplicons are sequenced and identified [16,19,21,22,23]. The sequences generated require bioinformatic analysis, and a ‘pipeline’ is designed for this purpose which processes the large data set through several stages: Quality control, where short or low confidence sequences are removed; dereplication, which aims to reduce the effects of PCR bias by selecting only one of each sequence; removal of chimeras, taking out of the equation sequences produced with amplification errors; and finally sequence identification, which is often conducted by searching online databases for matching published sequences, and thereby assigning a species or genus depending on the level of specificity in the results. This process results in a measure of relative abundance for each contributing sequence, which can be displayed in various ways, often a ‘heat map’ [19]. This is an advanced technique which in many ways represents the current ‘state-of-the-art’, but, as with all methods, it is only ever as reliable as the database to which it refers.

The abundance of available sequence information in public databases is an extreme benefit to the progress of DNA-based identification techniques; however, it does not come without pitfalls, as the number of incorrectly-labelled sequences demonstrates. This situation puts increasing importance on the knowledge and experience of the user, who must apply some criteria for acceptance to sequences acquired from databases before trusting their authenticity and cannot simply depend on the top BLAST result giving a confirmation of species. The use of DNA methods for the identification of a species is entirely dependent on the variation that can be measured between the ‘target’ species, and others that might be present. Therefore, the selection of sequences to differentiate between is a fundamental aspect of assay design. The incorporation of as much information as is available gives the greatest guarantee of specificity and, in order to achieve this, a curated dataset of validated sequences is required. These sequences may well be sourced from online facilities, but curation is essential to confer trust in the resource. The incorporation of sequences from the most closely related ‘sister’ species is the best guarantee of ‘future-proofing’ a method, as it can be deduced that sequences that differ in even the closest relative are likely to differ in all others also. However, as the redesign of this assay shows, the only true certainty can be gained by regular routine maintenance of methods—as is true for all techniques on the bench. For this work, a curated *Hypericum* database was created and used for the redesign of the qPCR method and also as one of the databases to interrogate with NGS data. 

The major benefits of both qPCR and NGS technologies have been described, but of course both methods also have drawbacks. For qPCR, the amount of information generated is much smaller and it is possible that unknown contaminants may not be picked up in the analysis. There is a requirement to standardise each reaction and measure efficiency, as every DNA extraction can behave differently. This has been achieved in several fields [24] but has not yet been applied to plant methods, which may well be more complex given their complicated genetic inheritance routes. For NGS metabarcoding methods, the effect of amplification biases must again be measured and controlled and this does not yet have a standard approach. Most importantly, metabarcoding is extremely similar to Sanger methods in its requirement for the whole barcode region to be present and not degraded. 

Many of these factors have been noted in publications [21], this is the first to conduct a direct comparison and provide a definitive answer to the question of what is most applicable to industry and regulators, and by doing so to aid in the production of high-quality herbal products for consumers.

## 2. Materials and Methods 

### 2.1. Sample Collection and Preparation

#### 2.1.1. Commercial Products

A range of different products sold as “St John’s Wort” (SJW) were purchased from high street shops and online suppliers. These included capsules, tablets and tea bags. Table 1 shows the full inventory of products along with the information provided on the packaging or information leaflets. A number of products carried the Traditional Herbal Registration (THR) logo which conveys a level of quality, whilst similar products were sold as food supplements. Sample number 222 is a mixture of five different plant species, but does not contain *H. perforatum*, and was used as a negative control. 

#### 2.1.2. Sample Preparation

Non-coated tablets were ground using a pestle and mortar before sampling. Coated tablets were cracked open and sample material taken from the middle to ensure the coating was not included. Capsule halves were separated and the contents emptied into a weighing boat and mixed manually using a spatula before sampling in order to obtain a representative sample. Individual tea bag contents were mixed and then a sample was powdered using a Tissue Lyser (Qiagen, Hilden, Germany). Samples of about 1 g of dried plant material were also powdered using a Tissue Lyser.

### 2.2. DNA Extraction

DNA extractions were carried out using the Qiagen DNeasy Plant Mini Kit according to the manufacturer’s instructions, starting with 0.02 g powdered plant or commercial product material. 

### 2.3. DNA Quantitation

The concentration of DNA in each extract was determined spectrophotometrically by measuring the A260 of a 2 µL aliquot using a Nanodrop Lite instrument (Thermo Scientific, Waltham, MA, USA). 

### 2.4. Conventional PCR and Gel Electrophoresis

PCR was performed using 1× MyTaq Red Mix (Bioline, London, UK), 0.2 µM of each forward (ITS1 5′-TCCGTAGGTGAACCTGCGG-3′) and reverse (ITS4 5′-TCCTCCGCTTATTGATATGC-3′) primers, and 1 µL of gDNA as template in a total reaction volume of 25 µL. Thermocycling conditions were optimized at 94 °C for 2 min, followed by 30 or 40 cycles of 94 °C for 15 s, 60 °C for 30 s and 72 °C for 30 s, with a final extension step of 72 °C for 2 min. PCR products were run on 2% (*w*/*v*) agarose, 1 x TBE gels with 1 µL SYBR^®^ Safe DNA Gel Stain (Invitrogen, Paisley, UK) at 100 V for 30 min and analysed in a Gel Doc™ EZ Gel Documentation System (BioRad, Oxford, UK). 

### 2.5. qPCR Assays with Specific & Generic Primer Pairs

#### 2.5.1. Protocol 1

qPCR assays consisted of EXPRESS SYBR^®^ GreenER™ qPCR SuperMix Universal (1×), relevant primers (0.1 µM each), and template DNA (1 µL) made up to a final volume 20 µL with sterile distilled water (SDW). All reactions were carried out in an MJ Research (Watertown, MA, USA) Chromo 4 real-time thermocycler. The primer pairs used for this assay were: HypGF (5′-CCGTGAACCATCGAGTCTTT-3′) with HypGR (5′-GTCTTACAACCACCGCTGGT-3′) and FO2 (5′-CATAAGAAGTGTAAGGCTCCCGG-3′) used with HRI-S (5′AGAGTCGTTATTGTTATGAACAGAAGGAG-3′). qPCR was performed using three biological replicates with three technical replicates for each sample. Water was run as a negative template control for each test. Thermocycling conditions were optimized at 2 min at 50 °C activation step, 2 min at 95 °C initial denaturation step, 40 cycles consisting of 15 s at 95 °C, 1 min at 60.1 °C, plate read, followed by a melt curve from 54–95 °C at a rate of +1 °C per 10 s, read every 2 °C.

#### 2.5.2. Protocol 2

Each qPCR assays contained 1× Sensifast SYBR green Hi-Rox mix (Bioline), 0.5 µL of gDNA or gBlock, 0.1 µM of each forward and reverse primer in a total volume of 10 µL made up with SDW. qPCR was performed using three biological replicates with three technical replicates for each sample.

Synthetic DNA molecules (gBlocks—IDT, Leuven, Belgium) corresponding to *H. perforatum* haplotype 1 (C178), *H. perforatum* haplotype 3 (C22), *H. maculatum* (C206) and *H. patulum* (C203) ITS sequences were constructed as reference standards (Supplementary Information, Database 1) and dissolved in water to a stock concentration (S) of 10 ng µL^−1^. Serial dilutions of the *H. perforatum* type 3 standard from S^−3^ to S^−7^ (10–0.001 pg µL^−1^) were performed to generate a standard curve. A working dilution of 0.1 pg µL^−1^ of the standard DNA extracts was used as a reference template. Water was run as a negative template control for each test. Thermocycling conditions were optimized at 95 °C for 2 min, followed by 40 cycles of 95 °C for 5 s and 30 s at the primer specific Ta. The melting curve was obtained by heating the amplified template from 65 to 95 °C increasing the temperature by 0.5 °C per cycle.

Primers used in protocol 2 experiments included HypGF, HypGR and FO2 (see Protocol 1) plus 460for (5′-TCG CAA GAG ACA ATC GGG AAT-3′), 460rev (5′-CCA TCC TAT TCC CGA TTG TCT CTT-3′) and 650rev (5′-GTC ACT TTG TGA GTG TTC GAT GTT-3′). 

#### 2.5.3. Analysis of qPCR Results 

Analyses were conducted according to MIQE guidelines [25]. Absolute DNA levels were calculated from the C22 synthetic DNA standard curve of Cq plotted against DNA amount. The proportion of *H. perforatum* DNA in each sample was determined by calculating the ratio of specific/total DNA using the generic primers as the “reference gene” and compared to the control sample (*H. perforatum* C22 dilution S^−5^) using the comparative (2^−ΔΔCt^) method [26]. 

### 2.6. NGS Methods

Barcode region amplifications were conducted using Hotstar Hifidelity polymerase (Qiagen) as per manufacturer’s instructions, and barcode primers with NGS priming site attachments as follows:

ITS1 region:

Forward: 5′-TCGTCGGCAGCGTCAGATGTGTATAAGAGACAGGGAAGKARAAGTCGTAACAAGG-3′

Reverse: 5′-GTCTCGTGGGCTCGGAGATGTGTATAAGAGACAGCGTTCAAAGAYTCGATGRTTC-3′

ITS2 region: 

Forward: 5′-TCGTCGGCAGCGTCAGATGTGTATAAGAGACAGATGCGATACTTGGTGTGAAT-3′

Reverse: 5′-GTCTCGTGGGCTCGGAGATGTGTATAAGAGACAGGACGCTTCTCCAGACTACAAT-3′ 

After initial barcode region amplification, PCR products were purified (AMPure, Agencourt) and pooled, and a library constructed using these based on the Illumina 16S Metagenomic Sequencing Library Preparation. Index PCR with Nextera XT Index Kit v2 SetC (Illumina, San Diego, CA, USA) was conducted using KAPA HiFi HotStart ReadyMix (Kapa Biosystems, Boston, MA, USA) as per manufacturer’s instructions. The PCR assays were cleaned using AMPure beads following the published protocol, with the following modifications: 1:1 PCR product: AMPure bead initial binding; elution in 10 mM Tris pH 8.0. The resulting libraries were quality controlled using SpectraMax Quickdrop, Qubit HS dsDNA kit and were then run on Bioanalyzer with a HS DNA chip, after being diluted to approximately 1 ng/µl. Using these findings, the libraries were normalised to 4 nM each, and were denatured and diluted following the standard 16S protocol. A 40% PhiX spike-in was included in the sequencing run to compensate for low library complexity, as per the protocol. The complete library was loaded onto a MiSeq v2 500 reagent cartridge and were sequenced 2 × 250 bp paired-end reads. 

### 2.7. Bioinformatics

#### 2.7.1. Curated Database of the ITS Region of Significant *Hypericum* Species

A small database of ITS sequences from significant *Hypericum* species was constructed (Supplementary Information, Database 2). Species were chosen if they matched at least one of three criteria:

1. Close relatives of *H. perforatum*.
2. Common in commercial trade as ornamental or medicinal plants.
3. Reported to be found as adulterants of *H. perforatum*.

ITS sequences from each target *Hypericum* species were identified in GenBank and “seed” sequences were chosen from recently published studies based on large collections of vouchered specimens. The seed sequence was used to identify similar sequences using Blast Explorer to produce a crude phylogenetic representation using BLAST scores and an estimate of the final multiple alignment length [27]. This initial screen allowed clusters of similar conspecific sequences to be identified, and for anomalous conspecific sequences on distant branches of the tree to be excluded from the dataset. Clustered conspecific sequences were aligned and a consensus sequence was constructed by majority voting at each polymorphic position. In small datasets, it was often possible to identify a “type” accession sequence which reflected all of the majority polymorphisms. In large species datasets where it was possible to clearly define different haplotypes, these were recorded separately (e.g., three *H. perforatum* types and two *H. maculatum* types were recognised). All sequences were trimmed to start at the first base of the ITS1 region and finish at the last base of the ITS2 region.

#### 2.7.2. Next-Generation Sequencing Amplicon Processing

The raw paired-end reads were trimmed and quality filtered using Trim galore! v. 0.3.3 [28], a wrapper tool around Cutadapt [29] and FastQC [30]. The minimum phred quality score required for further processing the reads was set to 28. The surviving reads were then further processed with USEARCH v. 9.2.64 [31]. Specifically, reads with identical sequences were reduced to a single read while keeping the number of contributing reads in the header. Sequences that were made up from less than 10 reads were discarded. In the next step, chimeric sequences were removed with the *-uchime2_denovo* algorithm [32,33]. The final steps consisted of summarising sequences with >0.99 identity to a single cluster. The remaining clusters were then separated into two different files, containing either the ITS1 or the ITS2 clusters.

The clusters were then queried to the nucleotide NCBI and the local database using nucleotide blast (blastn-megablast) in BLAST+ v. 2.2.26 [34]. The local database consisted of the *Hypericum* samples described above.

The resulting blast output files were then imported to MEGAN v. 5.11.3 for taxonomic profiling. The lowest common ancestor (LCA) parameters were set to minScore = 50, maxExpected = 0.01, topPercent = 3 and minSupport = 1. The taxonomic information and the number of reads contributing to a specific taxonomic hit was exported to a species-abundance table and further processed with custom R scripts and the R package ‘tidyverse’ [35]. In the case that the blast search against the NCBI database returns hits to several, potentially distant, taxa, the next deeper taxonomic level is returned. Some of these hits were very unspecific and resulted in very deep taxonomic levels, such as Pentapetalae. In this analysis, such categories were treated as “no hit”. The relative abundance of reads in each cluster was calculated relative to the total number of assigned reads per sample (including the reads treated as “no-hit”). Taxonomic categories that were less abundant than 2% were excluded from further analysis. The filtered dataset, containing the taxonomic categories, together with their relative abundance per sample were displayed in a heatmap. The heatmap was calculated for each region alone and as a combined dataset. In order to compare the NCBI blast results to the local BLAST search, the clusters which resulted in a hit to genus *Hypericum* or one of its species in the NCBI search were further investigated. The best matching taxon per cluster was extracted. This was based on the percent sequence identity (“pident”), rather than the e-value or the bit score.

## 3. Results

### 3.1. Isolation and Characterisation of DNA Extracted from Commercial Products

DNA samples extracted from a range of commercial capsules, tablets and tea bags were tested (Table 1). The amount of DNA extracted was quantified by measurement of A_260_ (Table 2). There was a considerable range of DNA quantities, ranging from undetectable to over 150 ng/mg.

**Figure 1:**
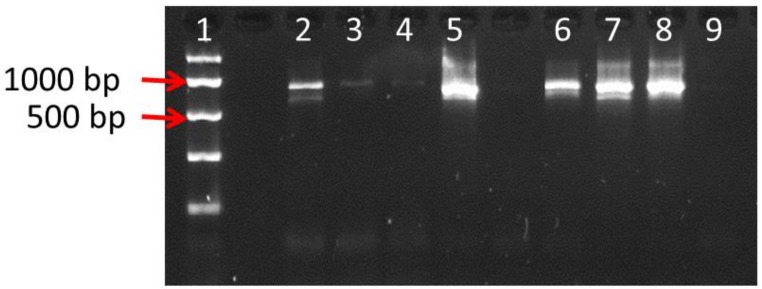
Agarose gel electrophoresis of PCRs using internal transcribed spacer 1 (ITS1) and ITS4 primers. Gel lanes: 1-Easy Ladder I (Bioline); 2-Positive control; 3-Sample 215; 4-Sample 215; 5-Sample 218; 6-Sample 224; 7-Sample 228; 8-Sample 229; 9-Negative (no template) control.

The quality and integrity of the DNA was determined by conventional PCR using the standard ITS barcode primers ITS1 and ITS4. Of the twenty products tested, five SJW samples (215, 218, 224, 228, 229) showed amplification of the full-length ITS barcode, as indicated by a positive band of the expected size following gel electrophoresis (Figure 1). There was some correlation between the success of the PCR and the concentration of DNA in the sample, but some samples with apparently high levels of DNA did not amplify (234, 244) whilst others with less DNA were successfully amplified (215, 218). The control sample 222 also produced an ITS band. These results are summarised in Table 2.

The remaining extracted samples were further purified using an iso-propanol clean-up procedure to remove potential PCR inhibitors. The effect of simple dilution (1:10) of the DNA extracts before PCR was also tested. However, neither of these procedures resulted in detectable amplification of the ITS region for any of the remaining samples, indicating that PCR inhibitory secondary products were not responsible for the poor template activity of the majority of the DNA extracts. 

The ITS amplicons from successful PCR assays were sequenced by conventional Sanger sequencing using the forward (ITS1) and reverse (ITS4) primers. Of the five SJW samples, only three (218, 228, 229) produced sufficiently abundant ITS amplicons to generate high quality sequence data (Figure 1). BLAST searching of GenBank with these sequences showed them all to have the highest scoring hits to *H. perforatum* ITS accessions with scores equivalent to >99% identity (Table 2). Two of these samples were tea bags containing dried leaf material (218 and 229), whilst the third was a capsule (228) containing a processed powder.

The remaining samples that could not be amplified with the ITS primers were tested for DNA integrity using *H. perforatum*-specific ITS primers (FO2/HRI-S) that produce a very short amplicon of 80 bp [13]. Conventional PCR with these primers yielded products that were difficult to distinguish from primer dimers by gel electrophoresis. For this reason, real-time PCR (using Protocol 1) was carried out as a more sensitive method for the detection of small amplicons. A pair of generic internal ITS primers (HypGF/HypGR, amplicon size ~220 bp) were also used to determine and measure the presence of DNA from any *Hypericum* species (Table 2).

The Cq values for the successful ITS amplified samples were, as expected, much lower than the other samples, with values in the range 16–20 cycles (Table 2). It was also noted that the Cq values using the generic and specific primer pairs were similar, confirming the ITS BLAST ID of this DNA as originating from *H. perforatum* (i.e., that the number of copies of *H. perforatum* ITS sequence was similar to the total number of ITS copies). In contrast, the negative control sample 222 gave a positive signal with the generic primers but not with the specific ones, confirming the presence of amplifiable “non-*H. perforatum*” plant DNA in this sample (Table 2). 

Two samples (226, 251) were noted as having Cq values for both primer pairs in the ranges 20–30 cycles, whilst the other three samples (232, 245, 250) had Cq values between 28–35 cycles with one or both pairs of primers. The melt curve profiles of these samples matched the positive control, indicating that the signal resulted from amplification of the expected product. The remainder of the samples had Cq values > 35 cycles, similar to the no-template controls. The melt curve profile of these samples did not show any evidence of the correct amplicon, but produced a peak with a lower melting temperature, indicative of primer dimer formation. The working conclusion from these results was that the five samples (215, 218, 224, 228, 229) that can act as templates for the ITS PCR (amplicons > 600 bp) contain reasonably intact DNA molecules. On the other hand, the five samples (226, 232, 245, 250, 251) that can only act as templates to produce short amplicons (<250 bp) most likely contain DNA fragments that are not long enough to produce sufficient, intact full-length ITS sequences.

The original five “intact ITS” samples plus the five “fragmented DNA” samples were therefore chosen for further analysis (Table 2).

### 3.2. New Primer Design for Specific PCR Testing

The detection of fragmented DNA in a number of commercial products raised several questions:

1. Is the detection of fragmented DNA supported by other assays?
2. Is it possible to identify the species of origin of these DNA fragments?
3. Could this information be used to authenticate processed herbal products?

One approach to answering these questions was to improve the real-time PCR assay. The results with the original *H. perforatum*-specific primer pair FO2/HRI-S described by Howard et al. [13] were found to be sub-optimal for two reasons:

1. The efficiency of these primers for qPCR was found to be poor, typically around 0.80.
2. The specificity of the primers was broader than could be known when they were first designed. There are many more *Hypericum* ITS sequences now in GenBank than were present before 2009, and a BLAST search of the database with the FO2 and HRI-S primers gives 100% matches with accessions from several *Hypericum* species. In addition, the primers were found not to be a perfect match to the *H. perforatum* ITS haplotype 1, so could give both false positive and false negative signals.

#### 3.2.1. Design of *H. perforatum*-Specific Primers

**Figure 2:**
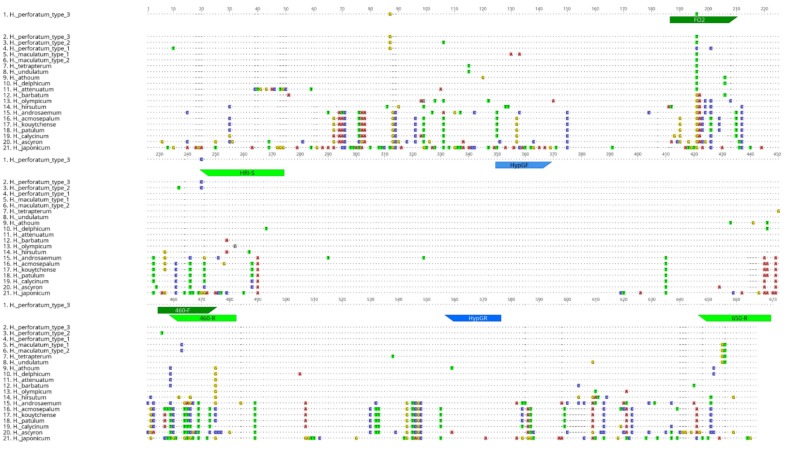
Multiple alignment of *Hypericum* ITS sequences showing the position of primers.

New qPCR primers were designed with a view to improving the efficiency and specificity of the PCR. The original design of the FO2/HRI-S primers was possible by manual inspection of an alignment of all the existing *Hypericum* ITS sequences available in 2008. Since then, the number of *Hypericum* sequences available has grown considerably as the data from a number of larger phylogenetic studies have been deposited in GenBank. One problem with this abundance of information is that the length and quality of accessions and the reliability of their original identification is inconsistent. As a result, a BLAST search of the database with a genuine *H. perforatum* ITS sequence will often include sequences from a number of different species in the top 50 hits, some having higher scores than genuine *H. perforatum* accessions. In order to filter this “background noise” effect, a smaller curated database of reliable primary sequences and derived consensus sequences was constructed (Figure 2). The database was also restricted to a small number of *Hypericum* species which meet some or all of the following criteria:

1. They are close relatives of *H. perforatum*.
2. They are common in commercial trade as ornamental or medicinal plants.
3. They have been reported to be found as adulterants of *H. perforatum* [9,10,15,36].

Since a test for commercial adulterants does not need to include rare *Hypericum* species that bear little resemblance to *H. perforatum*, the database of just twenty species included several close relatives from the Section *Hypericum* (*H. attenuatum*, *H. maculatum*, *H. tetrapterum*, *H. undulatum*), a number from the sections Ascyreia (*H. acmosepalum*, *H. calycinum*, *H. kouytchense*, *H. patulum*) and Adenosepalum (*H. athoum*, *H. delphicum*) and single representatives from the sections Androsaemum (*H. androsaemum*), Drosocarpum (*H. barbatum*), Roscyna (*H. ascyron*) Taenocarpum (*H. hirsutum*) and Trignobrathys (*H. japonicum*) [37]. 

Multiple alignment of sequences in the curated database allowed three regions to be identified where the *H. perforatum* ITS sequence showed the most variation from other *Hypericum* sequences (Figure 2). One of these, in the ITS1 region, contained the FO2 primer site, and this primer was still found to cover the area of highest variability in this region. The other two sites were found in the ITS2 region around positions 460 and 650 bp. Forward and reverse primer pairs were designed at the 460 bp site, giving three primer pairs: FO2-460rev, 460for-650rev and FO2-650rev. These are shown diagrammatically in Figure 2. The specificity of these primers was not unique to *H. perforatum* at each position. For example, FO2 matches the consensus sequences of all of the species from the section *Hypericum*. The forward and reverse primers at position 460 match *H. tetrapterum* and *H. undulatum*, whilst the primer at position 650 matches *H. attentuatum* and *H. undulatum*, but neither primer is a perfect match to the closest relative of *H. perforatum*, which is *H. maculatum*. 

The primers were initially screened for efficiency and specificity by conventional PCR using genomic DNA from several *H. perforatum* and non-*perforatum Hypericum* samples. The results show amplification of *H. perforatum* DNA (sample 1456), but no amplification of *H. patulum* DNA (sample 1479) by all three primer pairs following optimisation of the annealing temperatures of the PCRs (Supplementary Information, Figure S1). The strong amplification of both samples with the *Hypericum* generic primers shows the quality of both DNA templates (Supplementary Information, Figure S1D).

#### 3.2.2. Development of a Quantitative Assay for *H. perforatum* DNA Fragments

The efficiency of the three newly designed primer pairs was tested using the real-time PCR protocol 2 with a dilution series of the synthetic *H. perforatum* C22 standard template. The PCR efficiencies of the two primer pairs containing the FO2 primers were sub-optimal, with FO2-460rev having a very poor efficiency of 0.58 and FO2-650 one of 0.83. In contrast, the 460–650 primer pair had an efficiency of 0.98 and the HypG pair efficiency was 1.05 (Supplementary Information, Figure S2). The melting curve showed the presence of only specific products (Supplementary Information, Figure S3).

**Figure 3:**
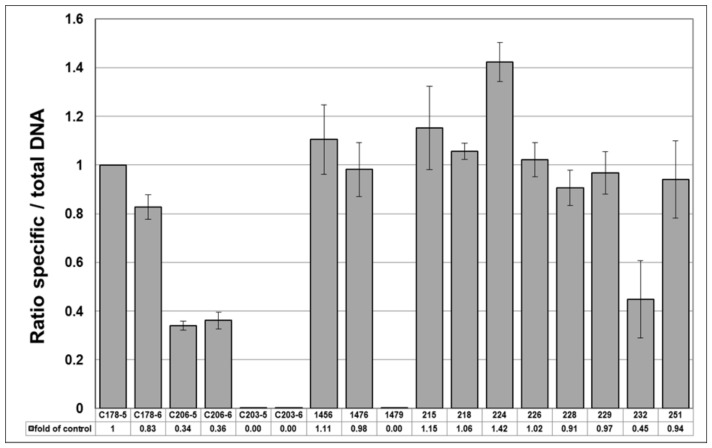
Ratio of specific *H. perforatum* DNA to total DNA as calculated from qPCR results of specific primers 460–650 compared to generic primers HypGF-HypGR. The ratios are calibrated to the gBlock standard C178-5. Two dilutions of each gBlock C178 (*H. perforatum*), C206 (*H. maculatum*) and C203 (*H. patulum*) are shown. Genomic DNA samples from plant specimens 1456, 1476 and 1479 and commercial samples 215–251 are shown.

The 460–650 pair was therefore chosen as the basis of a qPCR assay that could be used to determine the amount of DNA extracted from a processed medicine and the proportion of DNA copies that originate from *H. perforatum*. Two methods of analysis were used:

1. An absolute method of ITS sequence quantitation with the HypG primers using a standard curve of a synthetic template of known copy number. The synthetic *H. perforatum* standard C22 was used to create a standard curve by serial 10-fold dilutions over the range S^−3^–S^−8^ (10–0.0001 pg µL^−1^). Direct conversion of mean Cq value for each sample from the standard curve allowed the number of ITS copies to be calculated (Table 3). The three reference standards C178, C203 and C206 each showed very good correspondence between the copy numbers calculated for the 0.01 and 0.001 pg µL^−1^ dilutions.
2. The relative method of Pfaffl [38] was used to calculate the ratio of specific *H. perforatum* ITS sequences to total ITS sequences, with the generic HypG primers acting as the “reference gene” and the C178 standard used to calibrate the ratio to 1.0. As expected, the values for the two *H. perforatum* genomic DNA samples 1456 and 1476 were close to 1.0 (Table 3, Figure 3). The *H. patulum* standard C203 and genomic DNA 1479 gave ratios of 0.00, indicating that none of the ITS sequences present were from *H. perforatum*. However, the *H. maculatum* standard C206 showed a ratio of 0.37–0.39. This could be indicative of a significant cross-reaction with the 460–650 primers, suggesting that the reaction requires further optimisation to ensure that mismatch priming does not occur with these primers.

The qPCR assay was then used to test the commercial samples. The absolute determination of the ITS copy number with the HypG primers showed considerable variation across the samples, with the higher values typically found in the “intact DNA” samples (Table 4). Even within the “intact” group there were 10^−4^ fewer ITS copies in sample 215 than in sample 228. Of the “fragmented DNA” samples, ITS DNA was detected in samples 226 and 251 at levels higher than in 215. The levels of detection in the other three samples were much lower and in sample 250 was based on a Cq value of 34.68, close to the NTC value. Samples 232 and 245 had Cq values between 30 and 35 but showed the correct melt curve profile, indicating the presence of ITS DNA, but close to the limits of reliable detection.

Using the relative method to obtain the ratio of specific *H. perforatum* DNA to total *Hypericum* DNA, seven of the ten samples showed a reliable positive reaction with the 460–650 specific primer pair. Samples 245 and 250 had Cq values close to 35 from singleton readings and were considered unreliable, particularly when combined with the very low amount of detectable DNA in these samples. 

The specific/total ratios obtained for the remaining eight commercial samples are shown in Figure 3 along with the standards and plant genomic DNA results shown in Table 3. The ratios of the two *H. perforatum* genomic DNA extracts are close to 1.0, showing the expected result for a pure *H. perforatum* sample. Samples 215, 218, 226, 229 and 251 have ratios close to 1.0, indicating that these products contain *H. perforatum* DNA with very little contamination by other *Hypericum* species. The value for sample 224 is anomalously high, whilst samples 228 and 232 are significantly lower than 1.0. The ratio for sample 232 is 0.45, but the reliability of this value is questionable given the high Cq value with the specific 460–650 primer pair and the large errors bar shown Figure 3. The ratio of 0.91 for sample 228 is based on much lower Cq values and appears to be significantly below 1.0, indicating about 10% contamination by non-*H. perforatum* ITS sequences.

### 3.3. NGS Results

The original five “intact ITS” samples plus the five “fragmented DNA” samples that were selected for further qPCR analysis were also submitted for NGS metabarcoding analysis (Table 2). This began with an initial amplification of the ITS1 and ITS2 barcode regions, with primers extended to include NGS priming sites, resulting in a visible amplicon via gel electrophoresis for five out of the ten samples (218, 224, 228, 229, 251). Because of the lower detection levels possible with NGS methods, all ten samples were submitted for NGS analysis regardless of the presence of a visible band. However, only the five samples with bands were successfully analysed.

For the five successful samples, the total number of reads per sample in clusters varied from 63,271 to 162,586 and were in 121–469 clusters (Table 5). The water control had no sequence clusters, suggesting that contamination between samples or from external sources did not affect the results. The percent reads per sample that were not assigned ranged from 3.3 (sample 251) to 48.5 percent (sample 218). These reads matched the categories ‘cellular organisms’ (11%), ‘environmental sample’ (7.5%), ‘*Eukaryota*’ (33.5%), ‘*Pentapetalae*’ (47.9%) or were not assigned by the blast algorithm at all (<0.1%).

**Figure 4:**
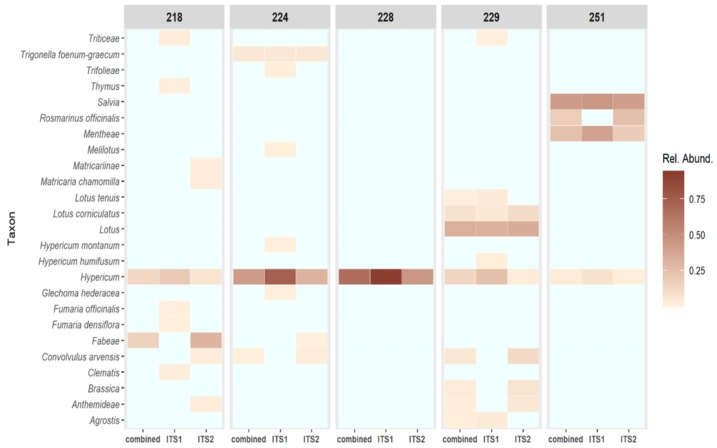
The heatmap displays the relative abundance of reads matching a given taxon after running BLAST against the NCBI database.

After quality filtering, the sequences were used to interrogate the NCBI database; using these data, the relative abundances of taxa were calculated and are shown as a heatmap in Figure 4. The total percentage of reads that matched exclusively to *Hypericum* ranged from 4.5–66.8 percent (Table 5), indicating that clear quality differences between the samples exist. Sample 218 is mainly contaminated with sequences from the Fabaceae subfamily Fabae. Sample 224 contains *Convolvulus arvensis* and *Trigonella foenum-graecum* sequences and sequences from the Fabaceae subfamily Trifoliae in addition to *Hypericum*. Sample 228 appears to only consist of *Hypericum* sequences, whereas contamination can be found in other samples. The species profile of sample 229 is rather complex and sequences from genera *Brassica* and *Agrostis* were found alongside a strong signal for *Lotus* sequences. Sample 251 exhibits contamination with *Rosmarinus officinalis*, genus *Salvia* and members of the Lamiaceae subfamily Menthae.

**Figure 5:**
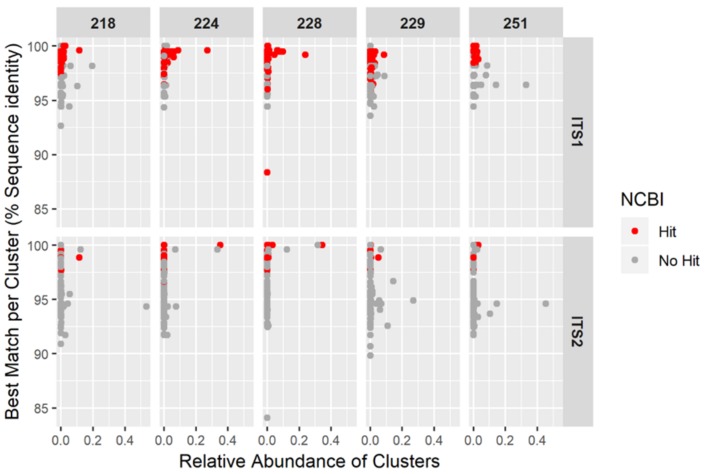
Result of searching the sequence clusters against the NCBI database with reference to results which are a ‘hit’ for *Hypericum* (red dots) and those that are not (grey dots). Each data point represents a sequence cluster and the colour denotes whether a cluster was assigned to genus *Hypericum*.

In order to assess the impact of using a curated *Hypericum* database to analyse the NGS sequences, they were first screened against NCBI. The sequences which did not match to any *Hypericum* species (shown in grey, Figure 5) were not taken forward for further analysis. The clusters shown in red (Figure 5) yielded results within the *Hypericum* genus from NCBI, and were therefore submitted for analysis against the curated database. As shown in Figure 5, the highest percent sequence similarity does not always correspond with the taxonomic identification of *Hypericum*, this is another representation of the sequences responsible for the heatmap ‘hits’ outside of the *Hypericum genus*.

**Figure 6:**
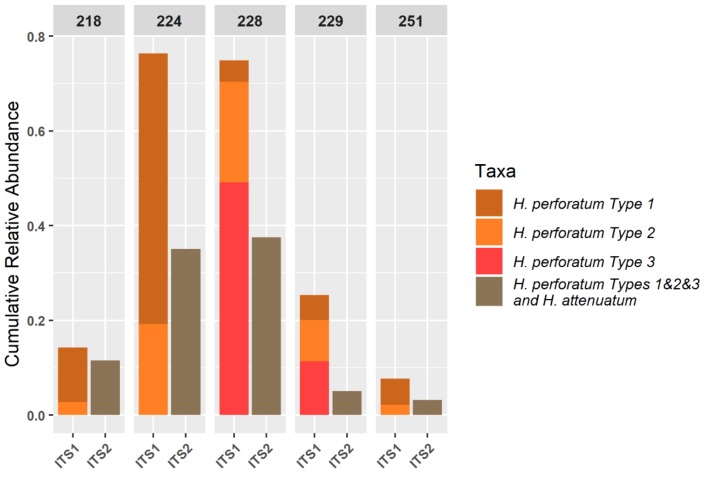
Taxonomic profiles of the clusters queried to the local *Hypericum* database. Only sequence clusters that matched genus *Hypericum* in the NCBI BLAST were used to further investigate the species identity.

The filtering of clusters matching *Hypericum* in the NCBI database and the subsequent analysis with the local blast search reveals that most of the ITS1 sequences match best to *H. perforatum* types 1, 2 or 3. Samples 224 and 228 gave strong positive results for *H. perforatum*, indicating a larger number of sequences in the initial DNA extraction and giving an indication of quantity. These two samples are made up of different types of *H. perforatum*, 224 is approximately ¾ type 1 and ¼ type 2 whereas sample 228 is predominantly type 3 with a smaller proportion made up of type 2 and very little type 1. The ITS2 region analysis is less nuanced and the local BLAST search resulted in equal matches to *H. perforatum* types and *H. attenuatum* (Figure 6); however, based on analysis of the database, this region would have picked up and *H. maculatum* sequences would have presented.

## 4. Discussion

The application of qPCR and NGS metabarcoding to discern identity and purity of herbal products was assessed using 19 samples from a variety of processed products claiming to contain St John’s Wort. Around half of these products were not suitable for either qPCR or NGS analysis due to the low yield and/or poor quality of DNA extracted from these materials. This is an anticipated finding, and relates directly to the composition of the products under analysis. The samples yielding the highest concentrations of amplifiable DNA are largely made up of plant material that has undergone minimal processing, such as the teas (samples 218 and 229). Conversely, many of the samples displaying low abundance of DNA are produced from extracts, and may well contain no plant material, such as samples 213 and 230—each tablet containing standardized extracts. In this situation the anomalies become of interest also, such as samples 235 and 244 that are labelled as containing a mass of ‘SJW’, but yield no DNA. The meaning of this label statement is not clear, and could equally refer to plant material or extract, and clarity on this may well be useful to the consumer. It is possible that techniques such as ‘baiting’ coupled with NGS would be capable of analysing these materials, but these results generally confirm the long-held view that molecular methods are not suitable for routine quality testing of processed extracts, and are better applied further up the supply chain, ideally focussing on raw materials, as described previously [19]. However, this study has adopted the workflow of a forensic investigation, using the initial FO2/HRI-S PCR assay as a presumptive test and three investigative tools (ITS barcoding, qPCR and NGS) to analyse those samples in which traces of DNA were detected. It is perfectly legitimate to apply these tools to determine the quality of market products provided that the underlying concepts of forensic investigation are fully understood. 

The remaining 10 samples fell into two distinct categories based on whether they produced templates that were suitable for NGS methodologies (Table 6). It is worth noting that the samples that produced NGS data had significantly more DNA and were less fragmented than those that did not. This suggests that a lower ‘cut-off’ limit of DNA concentration may be applicable to NGS metabarcoding methodologies, and further investigations to show exactly where these boundaries lie would be a useful addition to the field. There is a good correlation between the qPCR and NGS metabarcoding results for the five samples that produced both. They all had qPCR ratios of specific/total *Hypericum* DNA >0.90, consistent with the strong *Hypericum* signal in the NGS analysis. Samples 218 and 224 in particular had qPCR ratios >1.00 and good quality NGS profiles, consistent with the presence of good quality *H. perforatum* DNA. 

The qPCR results of samples 229 and 251 show ratios of 0.97 and 0.94, respectively (Table 6), which could indicate low levels of contamination by other *Hypericum* species. This is consistent with the NGS profiles, which show some contamination of both samples by a range of commercial herbal plant species (Figure 4) and a low relative abundance of identified *H. perforatum* sequences (Figure 6). It is likely that this is the result of cross-over in manufacturing processes, given the types of species present. It should be noted that the HypG primers are universal for ITS sequences of the *Hypericum* genus but may well not amplify and therefore measure the DNA that is picked up as background in the NGS method. The use of total plant generic primers targeting the conserved 5.8S region [39] would be a useful addition to this work. 

It has previously been claimed that NGS metabarcoding methods are too sensitive for herbal samples, which are by nature likely to contain low-level contamination below an acceptable threshold. However, using a logarithmic proportional scale displayed via a heatmap, this study has shown that even a finished product can give an ideal ‘pure’ result. However, more work is required to explore where the boundary of ‘acceptable’ should lie with respect to contamination. The novel use of a smaller, curated database to further profile sequences within the target genus yielded high specificity—showing the different types of *H. perforatum* present in each sample (Figure 6). The resolution of *H. perforatum* genotypes could be used to investigate the genetic basis of phytochemical variation in *H. perforatum* and would be extremely useful to companies for identifying and maintaining the quality of their sourced plant materials.

The laboratory methods and bioinformatics pipeline required to complete this analysis is significantly more demanding on the analyst than the qPCR method. This remains a barrier to entry for new users into this field and can prevent even university-based research groups from accessing NGS technology. As time and methods progress, this is likely to become less of an issue, but the added benefit of these methods for herbal drugs must be compelling in order to persuade industry to invest.

The approach used in both the original simple PCR test for *H. perforatum* and this improved and quantitative qPCR method represents an accessible route to entry into DNA quality control testing for industry with lower capital costs and, more importantly, less analytical skill required compared to NGS metabarcoding. The results also indicate that qPCR is more resilient to fragmented DNA samples than metabarcoding, as is to be expected from the nature of the methodologies, as the qPCR method utilises shorter fragments. However, even with the qPCR method less than half of the samples produced convincing results. 

NGS metabarcoding provides a significantly higher level of detail about the constituents of a DNA sample, giving information not only on the presence of ‘target’ species (*Hypericum* in this case) but also broadening the scope and identifying un-anticipated contaminants. This study has shown the utility of this, where the ‘panel’ of adulterants and contaminants was made up of *Hypericum* species, and therefore the qPCR test did not directly pick up contamination with distantly related herbal plants. The resolution offered by NGS metabarcoding enables the monitoring of supply chains and production methods and can provide clues as to the potential sources of contamination, for example crossing over from production equipment as discussed earlier. 

While there is debate around the application of molecular methods toward quantitative information on plant materials, qPCR methods have been applied in this respect to many other areas such as GMOs, food and viral load. The question of this application relates back to the point in the supply chain where sampling occurs and would be achievable when analysing raw materials. For this to be validated, standards would be required for each qPCR, as the DNA matrix can have an effect on the reaction, which would confound results if unmonitored. The standards used to calibrate this method could be further adapted to provide such a reference, allowing standardisation of the technique. The question of quantitative validation is more problematic in the NGS metabarcoding scenario due to its broad scope, which is also its major benefit. If all species are the ‘target’ for the method, then considerable sequence variation can occur between ‘universal’ priming sites. This means that problems of preferential amplification are likely to occur, and, for example, shorter ITS regions, such as those in fungal genomes, could become misleadingly abundant due to the propensities of methods required for library construction. 

Many studies have recently underlined the need for better quality control and traceability of herbal products in the chain from field to food in all the continents [19,22,40,41,42,43]. DNA-based testing using qualitative PCR is already the prevailing method used for the identification of foodborne pathogens and for assuring food safety and, as recently suggested by Newmaster et al. (2019), this method delivers precise, sensitive, specific and reliable results. Newmaster et al. (2019) recommend that quantitative PCR DNA-based assays should be used to test plant material available on the market throughout the supply chain to verify and quantify possible adulterations and contaminations. Recent studies have also proved that DNA metabarcoding is a valid qualitative analytical approach to investigating discrepancies between products and products’ labels in complex multi-ingredient and processed mixtures [44].

The more reductionist a technique, the more vulnerable it is to the acquisition of new information. For this reason, the most applicable industry methods, with the most simple and easy approach must be iterative and change over time. Maintenance is key to preserving the integrity of any assay, allowing for previously unsampled natural variation and investigation of ‘Out of Spec’ (OOS) results. These findings must then be fed back into the system, generating a manageable maintenance routine for analysis. Weighing up the benefits and limitations of the two techniques investigated, the most efficient proposal for this is to employ routine qPCR techniques coupled with NGS metabarcoding for the understanding of OOS results. This would enable the redesign of the qPCR based on the known contaminants, as found over time using NGS metabarcoding.

The power and utility of DNA-based methods for the identification of herbal drugs is in its direct measurement of species, whereas other methods focus on secondary processes within the plant which are not fixed. However, the most efficient use of this information may be in increasing the stringency of other analytical methods. By designing high quality phytochemical or physical testing methods around materials that have been identified using DNA-based methods, the boundaries for ‘pass’ or ‘fail’ can be made more rigorous and can ensure that all unacceptable variation is ruled out. The inclusion of all methods and information for the design of quality control methods is fundamental; only by analysing all features can the most important and measurable discerning features be understood, and for medicinal plants molecular identity is an essential part of this picture.

## Figures and Tables

**Table 1:** Sample information and amount of material used in DNA extraction.

Sample Number	Type	Amount/Unit	Drug Extract Ratio and Information	THR
211	Tab	425 mg extract	5–7:1 (equivalent to 2135–2975 mg SJW). 60% Ethanol extraction.	Y
213	Tab	334 mg extract	5–7:1 (equivalent to 1670–2338 mg SJW). 60% Ethanol extraction.	Y
215	Cap	142 mg extract	Equivalent to 711–995 mg SJW. 60% Ethanol extraction.	Y
218	Tea bag	1.5 g SJW		
221	Cap	450 mg	Standardised to 0.3% hypericin (1.35 mg)	
223	Tab	500 mg SJW powder	Standardised to 0.3% total hypericins	
224	Cap	200 mg SJW powder	(Brown rice flour filler stated)	
226	Cap	300 mg	Standardised to 0.3% hypericin (0.9 mg)	
228	Cap	500 mg SJW	0.02% hypericin	
229	Tea bag	2 g SJW		
230	Tab	425 mg extract	3.5–6:1 (equivalent to 1490–2550 mg SJW). 60% ethanol extraction.	Y
232	Tab	40–73 mg extract	3.1–4.0:1. 60% ethanol extraction.	Y
233	Tab	333 mg	Standardised to 0.3% hypericin (1000 µg)	
234	Tab	1000 mg	5:1 = 5000 mg dried leaf	
			3000 µg hypericin	
235	Tab	300 mg SJW		
244	Cap	350 mg SJW		
245	Cap	300 mg	Standardised to 0.3% hypericin (0.9 mg)	
250	Cap	300 mg	Standardised to 0.3% hypericin (0.9 mg)	
251	Tab	315 mg powder	Standardised to 0.3% hypericin	
222	Tab	34 mg motherwort extract	*Leonorus cardiac*, *Humulus lupulus*, *Passifloroa incarnata*, *Lactuca virosa*, *Valeriana officinalis*. 60% ethanol extraction.	

**Table 2:** Traditional barcoding, and qPCR results. Traditional barcoding results describe the presence of a detectable band after gel electrophoresis: - not detectable, + detectable, ++ strongly detectable. For those bands that provided sequence information, the top result of a BLAST search is shown. Samples marked with * were deemed as not suitable for further DNA based analysis, having given no results in traditional PCR and generic qPCR testing methods. Cq results are shown for the original primers used in qPCR, rounded up to the nearest integer.

Sample Number	ng DNA per mg Sample	Traditional Barcoding		qPCR Cq Values	
		ITS Band	ITS BLAST ID	HypGF/HypGR	FO2/HRI-S
211 *	21.0	-		35	35
213 *	14.5	-		37	35
215	37.5	+		35	34
218	36.5	++	*H. perf* (99%)	18	17
221 *	18.5	-		34	30
223 *	23.0	-		37	37
224	29.6	+		19	17
226	13.5	-		27	25
228	158.5	++	*H. perf* (99%)	16	17
229	100.5	++	*H. perf* (99%)	16	16
230 *	44.5	-		35	34
232	0.0	-		32	31
233 *	108.0	-		38	N/A
234 *	160.5	-		N/A	N/A
235 *	46.5	-		37	N/A
244 *	150.0	-		N/A	N/A
245	3.0	-		37	33
250	3.5	-		28	N/A
251	1.5	-		28	25
222	120.5	+	Mixed sample	28	36

**Table 3:** Standards for second generation qPCR design. The mean Cq and standard deviations (SD) were calculated from triplicate reactions containing synthetic reference standards (Std) and plant genomic DNA (plant) extractions. The number of ITS copies/µL were calculated from a standard curve of dilutions of the C22 reference standard plotted against Cq values using the HypG primers. The ratio of specific to total ITS copies was calculated directly using the formula of Pfaffl [38]. Spec/tot = specific/total.

Sample Number	Sequence Origin	Type	460–650	SD	HypG	SD	ITS Copies/µL × 10^3^	Ratio Spec/Tot
C178-5	*H. perforatum* (type 1)	Std	19.96	0.017	17.43	0.022	31.3	1.00
C178-6		Std	23.84	0.068	20.87	0.084	3.2	0.83
C206-5	*H. maculatum*	Std	20.27	0.069	16.22	0.047	69.6	0.34
C206-6		Std	23.95	0.176	19.82	0.078	6.4	0.36
C203-5	*H. patulum*	Std	35.8	1.748	16.77	0.503	50.4	0.00
C203-6		Std	>40	ND	20.18	0.082	5.1	0.00
1456	*H. perforatum*	Plant	16.27	0.108	14.03	0.234	296.8	1.11
1476	*H. perforatum*	Plant	13.68	0.025	11.40	0.152	1692.1	0.98
1479	*H. patulum*	Plant	29.65	0.329	12.60	0.053	764.1	0.00

**Table 4:** Testing of commercial samples using the updated qPCR assay. The mean Cq and standard deviation (SD) were calculated from triplicate reactions using the HypG and 460–650 primers are shown. The number of ITS copies/µL were calculated from the *H. perforatum* C22 reference standard curve. Cq values marked * indicate failure of one or more of the triplicate readings and a mean Cq value similar to the NTC control. The ratio of specific to total ITS copies was calculated directly using the formula of Pfaffl [38].

Sample Number	460–650	SD	HypG	SD	ITS Copies/µL × 10^3^	Ratio Spec/Tot
215	29.70	0.248	26.92	0.094	0.059	1.15
218	19.86	0.075	17.41	0.031	31.7	1.06
224	19.30	0.080	17.29	0.055	34.2	1.42
226	25.85	0.147	23.08	0.175	0.747	1.02
228	15.42	0.110	12.95	0.009	603.0	0.91
229	20.79	0.130	18.17	0.061	19.2	0.97
232	35.14	0.416	30.75	0.425	0.005	0.45
245	34.59 *	0.011	32.60	1.679	0.002	ND
250	36.19 *	ND	34.68 *	3.452	0.001	ND
251	23.92	0.172	21.11	0.103	2.74	0.94

**Table 5:** The total number of filtered and clustered reads per sample as analysed by metabarcoding. The number of clusters and the percent of ‘not assigned’ reads are summarised, and the percentage of *Hypericum* reads per sample shown.

Sample	Region	Total Reads in Clusters	Number of Clusters	Reads per DNA Barcode Not Assigned (%)	Reads per Sample Not Assigned (%)	*Hypericum* Reads per Sample (%)
218	ITS1	147,659	243	54.80	0.49	14.22
	ITS2	162,586	134	42.87		
224	ITS1	63,271	147	2.54	0.34	44.27
	ITS2	112,617	257	45.73		
228	ITS1	133,583	333	1.12	0.26	67.93
	ITS2	158,767	469	46.34		
229	ITS1	154,781	260	8.28	0.10	16.42
	ITS2	150,085	217	12.63		
251	ITS1	75,880	121	2.24	0.03	4.34
	ITS2	58,531	61	3.71		

**Table 6:** Correlation between DNA yield and qPCR and Next Generation Sequencing (NGS) results for the ten highest quality DNA samples. The success of the specific qPCR and NGS assays is related to the number of amplifiable ITS copies in the DNA extract. The quality of the samples determined by specific qPCR assay and NGS is also compared.

Sample Number	qPCR Ratio Specific: Total	NGS Results	ITS Copies/µL × 10^3^
215	1.15	n/a	0.059
226	1.02	n/a	0.747
232	0.45	n/a	0.005
245	n/a	n/a	0.002
250	n/a	n/a	0.001
218	1.06	Good quality, some background	31.7
224	1.42	Good quality, some background	34.2
228	0.91	Pure	603.0
229	0.97	Some *Hypericum*—strong Lotus signal	19.2
251	0.94	Some *Hypericum*—strong Salvia, Menthae and Rosmarinus signals	2.74

## Supplementary Materials

The following are available online at https://www.mdpi.com/2073-4425/10/4/286/s1, Supplementary Information, Database 1: Synthetic DNA molecules (gBlocks—IDT, Leuven, Belgium) corresponding to *H. perforatum* haplotype 1 (C178), *H. perforatum* haplotype 3 (C22), *H. maculatum* (C206) and *H. patulum* (C203); ITS sequences used as reference standards. Supplementary Information, Database 2: Database of ITS sequences from significant *Hypericum* species, Figure S1: Agarose gel electrophoresis of PCRs showing specificity of the new generation primers, Figure S2: Quantitative Real-time PCR standard curve *Hypericum perforatum* serial dilutions with the (A) generic HypG F/R primers and the (B) specific 460F-650R primers, Figure S3: Quantitative Real-time PCR Melting curve generated with the (A) generic HypG F/R primers and the (B) specific 460F-650R primers.
