# Supplementary material for: DNA Authentication of St John’s Wort (Hypericum perforatum L.) Commercial Products Targeting the ITS Region
Source: Genes (Basel). 2019 Apr 9;10(4):286. doi: 10.3390/genes10040286 (PMC6523358; doi:10.3390/genes10040286)
Supplement: Supplementary file 1 [file genes-10-00286-s001.pdf]

Supplementary Information, Database 1: Synthetic DNA molecules (gBlocks - IDT, Leuven, Belgium) corresponding to *H. perforatum* haplotype 1 (C178), *H. perforatum* haplotype 3 (C22), *H. maculatum* (C206) and *H. patulum* (C203) ITS sequences used as reference standards.

Name - Hypericum p C22 ITS — gBlocks® Gene Fragments — 727 base pairs

5'- GGT TTC CGT AGG TGA ACC TGC GGA AGG ATC ATT GTC GAA ACC TCC AAA TGA CCC GCG AAC TAG TTA TCC
ACA AGT CGG GGG TGT CGT GGG CTT CTG TCC CGG CGC CCC CGT GGC GGT GGT GGC CAG GCG CGC CAA GCT CTT
GGC ACG GCT GGC CCA TCA CCT GCC CAA CAA ACA AAC CCC GGC GCG GCA CGC GCC AAG GAA CTT TTG CAT CAT
AAG AAG TGT AAG GCT CCC GGC TGT GCC GGA AAT CGG ACA ACA CGG TCG GGG GCC TCC TTC TGT TCA TAA CAA
TAA CGA CTC TCG GCA ACG GAT ATC TAG GCT CTT GCA TCG ATG AAG AAC GTA GCG AAA TGC GAT ACT TGG TGT
GAA TTG CAG AAT CCC GTG AAC CAT CGA GTC TTT GAA CGC AAG TTG CGC CCG AAG CCT TCT GGC CGA GGG CAC
GCC TGC CTG GGT GTC ACA CAT CGT CGC CCC CCA AAA TCC CGA TAT CTC GCA AGA GAC AAT CGG GAA TAG GAT
GGG CGG AAA ATG GTC TCC CGT GCG CTC CCG TTC GCG GTT GGC CCA AAA ATG AGT TCC TGG CAA AGC AAA GCC
ACG ACC AGC GGT GGT TGT AAG ACC CTC GGT ACA AGT CGT GAG CCT TGC ATT GCT CGT AGG GAC ATG TTG ACC
CTG AAC GTG ATC GAG TAA CAT CGA ACA CTC ACA AAG TGA CCC CAG GTC AGG CGG GAC TAC CCG CTG AAT TTA
AGC ATA TCA A -3'

Note 1: The sequence information of the sense strand displayed above is intentionally truncated to the first 1,200 bases. The complete sequence can be verified in FASTA format in your order history.
Note 2: gBlocks® Gene Fragments are delivered as double-stranded DNA. Conformance to quality standards is established in multiple ways, including size verification by capillary electrophoresis and sequence identification by mass spectrometry.

Name - Hypericum p C203 ITS — gBlocks® Gene Fragments — 713 base pairs

5'- TTT TAA AGA GAG AAG AAA TAC AAG GTT TCC GTA GGT GAA CCT GCG GAA GGA TCA TTG TCG AAA CCT CCA
AAT GAC CCG CGA ACC AGT TAT CCA CAA GTC GGG GGT GTC GGG CTG AAC CCC GTA ACC CCC GTG CGC CGG TGG
CGG TCA GGC GTG CCA AGC TCT TGG CAC GGT TGG CCC GTC ACC TGC CCA ACA AAC CAA CCC CGG CGC GGC ACG
CGC CAA GGA ACT TTG CAT CAT GAG AAG GAC AAT GCC CCC GTC CGT GCC GGA AAT CGG ATA ACA CGG CCG GTG
GCT TTC CTT CTG TTC ATA ACT AAA ACG ACT CTC GGC AAC GGA TAT CTA GGC TCT TGC ATC GAT GAA GAA CGT
AGC GAA ATG CGA TAC TTG GTG TGA ATT GCA GAA TCC CGT GAA CCA TCG AGT CTT TGA ACG CAA GTT GCG CCC
GAA GCC TTC TGG CCG AGG GCA CGT CTG CCT GGG TGT CAC ACA TCG TCG CCC CCA AAA CCA ATG CCT CAC TCG
AGT TCA TTG GGT ACA GGA TGG GCG GAT AAT GGT CTC CCG TGC GCA CCC GTT CGC GGT TGG CCC AAA ACT TTG
TTC CTG GCG ATC GCA AGC CAT GAC CAG CGG TGG TTG TAA GAC CCT CGG TCA TAG TCG TGA GCT TGC ACG TCG
GGA CAT ATC GAC CCT GAA CGT GAT CGA GAA ACC TCG AAC ACT CAC AAA GTG ACC TCA GTC AGG GAT CC -3'

Note 1: The sequence information of the sense strand displayed above is intentionally truncated to the first 1,200 bases. The complete sequence can be verified in FASTA format in your order history.
Note 2: gBlocks® Gene Fragments are delivered as double-stranded DNA. Conformance to quality standards is established in multiple ways, including size verification by capillary electrophoresis and sequence identification by mass spectrometry.

Name - Hypericum m C206 ITS — gBlocks® Gene Fragments — 748 base pairs

5'- TAC AAG GTT TCC GTA GGT GAA CCT GCG GAA GGA TCA TTG TCG AAA CCT CCA AAT GAC CCG CGA ACT AGT
TAT CCA CAA GTC GGG GGT GTC GTG GGC TTC TGT CCC GGC GCC CCC GTG CCG GTG GTG GCC AGG CGC GCC AAG
CTC TTG GCA CGG CTG GCA CAA CAC CTG CCC AAC AAA CAA ACC CCG GCG CGG CAC GCG CCA AGG AAC TTT TGC
ATC ATA AGA AGT GTA AGG CTC CCG GCT GTG CCG GAA ATC GGA CAA CAC GGT CGG GGG CTT CCT TCT GTT CAT
AAC AAT AAC GAC TCT CGG CAA CGG ATA TCT AGG CTC TTG CAT CGA TGA AGA ACG TAG CGA AAT GCG ATA CTT
GGT GTG AAT TGC AGA ATC CCG TGA ACC ATC GAG TCT TTG AAC GCA AGT TGC GCC CGA AGC CTT CTG GCC GAG
GGC ACG CCT GCC TGG GTG TCA CAC ATC GTC GCC CCC CAA AAT CCC GAT ATC TCG CAA GAC ACA ATC GGG AAT
AGG ATG GGC GGA AAA TGG TCT CCC GTG CGC TCC CGT TCG CGG TTG GCC CAA AAA TGA GTT CCT GGC AAA GCA
AAG CCA CGA CCA GCG GTG GTT GTA AGA CCC TCG GTA CAA GTC GTG AGC CTT GCA TTG CTC GTA GGG ACA TGT
TGA CCC TGA ACG TGA TCG AGT AAC ATC GGT ACA CTC ACA AAG TGA CCC CAG GTC AGG CGG GAC TAC CCG CTG
AAT TTA AGC ATA TCA ATA AGG CGG GAG GAA A -3'

Note 1: The sequence information of the sense strand displayed above is intentionally truncated to the first 1,200 bases. The complete sequence can be verified in FASTA format in your order history.
Note 2: gBlocks® Gene Fragments are delivered as double-stranded DNA. Conformance to quality standards is established in multiple ways, including size verification by capillary electrophoresis and sequence identification by mass spectrometry.

Name - Hypericum p C178 ITS — gBlocks® Gene Fragments — 727 base pairs

5'- GGT TTC CGT AGG TGA ACC TGC GGA AGG ATC ATT GTC GAA ACC TCC AAA TGA CCC GCG AAC TAG TTA TCC
ACA AGT CGG GGG CGT CGT GGG CTT CTG TCC CGG CGC CCC CGT GGC GGT GGT GGC CAG GCG CGC CAA GCT CTT
GGC ACG GCT GGC TCA TCA CCT GCC CAA CAA ACA AAC CCC GGC GCG GCA CGC GCC AAG GAA CTT TTG CAT CAT
AAG AAG CGT AAC GCT CCC GGC TGT GCC GGA AAT CGG ACA ACA CGG TCG GGG GCT TCC TTC TGT TCA TAA CAA
TAA CGA CTC TCG GCA ACG GAT ATC TAG GCT CTT GCA TCG ATG AAG AAC GTA GCG AAA TGC GAT ACT TGG TGT
GAA TTG CAG AAT CCC GTG AAC CAT CGA GTC TTT GAA CGC AAG TTG CGC CCG AAG CCT TCT GGC CGA GGG CAC
GCC TGC CTG GGT GTC ACA CAT CGT CGC CCC CCA AAA TCC CGA TAT CTC GCA AGA GAC AAT CGG GAA TAG GAT
GGG CGG AAA ATG GTC TCC CGT GCG CTC CCG TTC GCG GTT GGC CCA AAA ATG AGT TCC TGG CAA AGC AAA GCC
ACG ACC AGC GGT GGT TGT AAG ACC CTC GGT ACA AGT CGT GAG CCT TGC ATT GCT CGT AGG GAC ATG TTG ACC
CTG AAC GTG ATC GAG TAA CAT CGA ACA CTC ACA AAG TGA CCC CAG GTC AGG CGG GAC TAC CCG CTG AAT TTA
AGC ATA TCA A -3'

Note 1: The sequence information of the sense strand displayed above is intentionally truncated to the first 1,200 bases. The complete sequence can be verified in FASTA format in your order history.
Note 2: gBlocks® Gene Fragments are delivered as double-stranded DNA. Conformance to quality standards is established in multiple ways, including size verification by capillary electrophoresis and sequence identification by mass spectrometry.

Supplementary Information Database 2: Database of ITS sequences from significant *Hypericum* species.

H._maculatum_type_1

TCGAAACCTCCAAATGACCCGCGAACTAGTTATCCACAAGTCGGGGGTGTCGTGGGCTTC

TGTCCCGGCGCCCCCGTGCCGGTGGTGGCCAGGCGCGCCAAGCTCTTGGCACGGCTGGCA

CAACACCTGCCCAACAAACAAACCCCGGCGCGGCACGCGCCAAGGAACTTTTGCATCATA

AGAAGTGTAAGGCTCCCGGCTGTGCCGGAAATCGGACAACACGGTCGGGGGCTTCCTTCT

GTTCATAACAATAACGACTCTCGGCAACGGATATCTAGGCTCTTGCATCGATGAAGAACG

TAGCGAAATGCGATACTTGGTGTGAATTGCAGAATCCCGTGAACCATCGAGTCTTTGAAC

GCAAGTTGCGCCCGAAGCCTTCTGGCCGAGGGCACGCCTGCCTGGGTGTCACACATCGTC

GCCCCCCAAAATCCCGATATCTCGCAAGACACAATCGGGAATAGGATGGGCGGAAAATGG

TCTCCCGTGCGCTCCCGTTCGCGGTTGGCCCAAAAATGAGTTCCTGGCAAAGCAAAGCCA

CGACCAGCGGTGGTTGTAAGACCCTCGGTACAAGTCGTGAGCCTTGCATTGCTCGTAGGG

ACATGTTGACCCTGAACGTGATCGAGTAACATCGGTACACTCACAAA

>H._maculatum_type_2

TCGAAACCTCCAAATGACCCGCGAACTAGTTATCCACAAGTCGGGGGTGTCGTGGGCTTC

TGTCCCGGCGCCCCCGTGCCGGTGGTGGCCAGGCGCGCCAAGCTCTTGGCACGGCTGGCC

CATCACCTGCCCAACAAACAAACCCCGGCGCGGCACGCGCCAAGGAACTTTTGCATCATA

AGAAGTGTAAGGCTCCCGGCTGTGCCGGAAATCGGACAACACGGTCGGGGGCTTCCTTCT

GTTCATAACAATAACGACTCTCGGCAACGGATATCTAGGCTCTTGCATCGATGAAGAACG

TAGCGAAATGCGATACTTGGTGTGAATTGCAGAATCCCGTGAACCATCGAGTCTTTGAAC

GCAAGTTGCGCCCGAAGCCTTCTGGCCGAGGGCACGCCTGCCTGGGTGTCACACATCGTC

GCCCCCCAAAATCCCGATATCTCGCAAGACACAATCGGGAATAGGATGGGCGGAAAATGG

TCTCCCGTGCGCTCCCGTTCGCGGTTGGCCCAAAAATGAGTTCCTGGCAAAGCAAAGCCA

CGACCAGCGGTGGTTGTAAGACCCTCGGTACAAGTCGTGAGCCTTGCATTGCTCGTAGGG

ACATGTTGACCCTGAACGTGATCGAGTAACATCGGTACACTCACAAA

>H._perforatum_type_2

TCGAAACCTCCAAATGACCCGCGAACTAGTTATCCACAAGTCGGGGGTGTCGTGGGCTTC

TGTCCCGGCGCCCCCGTGGCGGTGGTGGCCAGGCGTGCCAAGCTCTTGGCACGGCTGGCC

CATCACCTGCCCAACAAACAAACCCCGGCGCGGCACGCGCCAAGGAACTTTTGCATCATA

AGAAGTGTAAGGCTCCCGGCTGTGCCGGAAATCGGACAACACGGTTGGGGGCCTCCTTCT

GTTCATAACAATAACGACTCTCGGCAACGGATATCTAGGCTCTTGCATCGATGAAGAACG

TAGCGAAATGCGATACTTGGTGTGAATTGCAGAATCCCGTGAACCATCGAGTCTTTGAAC

GCAAGTTGCGCCCGAAGCCTTCTGGCCGAGGGCACGCCTGCCTGGGTGTCACACATCGTC

GCCCCCCAAAATCCCGATATCTTGCAAGAGACAATCGGGAATAGGATGGGCGGAAAATGG

TCTCCCGTGCGCTCCCGTTCGCGGTTGGCCCAAAAATGAGTTCCTGGCAAAGCAAAGCCA

CGACCAGCGGTGGTTGTAAGACCCTCGGTACAAGTCGTGAGCCTTGCATTGCTCGTAGGG

ACATGTTGACCCTGAACGTGATCGAGTAACATCGAACACTCACAAA

>H._perforatum_type_3

TCGAAACCTCCAAATGACCCGCGAACTAGTTATCCACAAGTCGGGGGTGTCGTGGGCTTC

TGTCCCGGCGCCCCCGTGGCGGTGGTGGCCAGGCGCGCCAAGCTCTTGGCACGGCTGGCC

CATCACCTGCCCAACAAACAAACCCCGGCGCGGCACGCGCCAAGGAACTTTTGCATCATA

AGAAGTGTAAGGCTCCCGGCTGTGCCGGAAATCGGACAACACGGTCGGGGGCCTCCTTCT

GTTCATAACAATAACGACTCTCGGCAACGGATATCTAGGCTCTTGCATCGATGAAGAACG

TAGCGAAATGCGATACTTGGTGTGAATTGCAGAATCCCGTGAACCATCGAGTCTTTGAAC

GCAAGTTGCGCCCGAAGCCTTCTGGCCGAGGGCACGCCTGCCTGGGTGTCACACATCGTC

GCCCCCCAAAATCCCGATATCTCGCAAGAGACAATCGGGAATAGGATGGGCGGAAAATGG

TCTCCCGTGCGCTCCCGTTCGCGGTTGGCCCAAAAATGAGTTCCTGGCAAAGCAAAGCCA

CGACCAGCGGTGGTTGTAAGACCCTCGGTACAAGTCGTGAGCCTTGCATTGCTCGTAGGG

ACATGTTGACCCTGAACGTGATCGAGTAACATCGAACACTCACAAA

>H._perforatum_type_1

TCGAAACCTTCAAATGACCCGCGAACTAGTTATCCACAAGTCGGGGGTGTCGTGGGCTTC

TGTCCCGGCGCCCCCGTGGCGGTGGTGGCCAGGCGCGCCAAGCTCTTGGCACGGCTGGCC

CATCACCTGCCCAACAAACAAACCCCGGCGCGGCACGCGCCAAGGAACTTTTGCATCATA

AGAAGCGTAACGCTCCCGGCTGTGCCGGAAATCGGACAACACGGTCGGGGGCTTCCTTCT

GTTCATAACAATAACGACTCTCGGCAACGGATATCTAGGCTCTTGCATCGATGAAGAACG

TAGCGAAATGCGATACTTGGTGTGAATTGCAGAATCCCGTGAACCATCGAGTCTTTGAAC

GCAAGTTGCGCCCGAAGCCTTCTGGCCGAGGGCACGCCTGCCTGGGTGTCACACATCGTC

GCCCCCCAAAATCCCGATATCTCGCAAGAGACAATCGGGAATAGGATGGGCGGAAAATGG

TCTCCCGTGCGCTCCCGTTCGCGGTTGGCCCAAAAATGAGTTCCTGGCAAAGCAAAGCCA

CGACCAGCGGTGGTTGTAAGACCCTCGGTACAAGTCGTGAGCCTTGCATTGCTCGTAGGG

ACATGTTGACCCTGAACGTGATCGAGTAACATCGAACACTCACAAA

>H._barbatum

TCGAAACCTCCAAATGACCCGCGAACTAGTTATCCACAAGTCAGGGGTGTCGTGGGCTTC

TGTCCCGGCGCCCCCGTGCCGGTGGTGGCCAGGCGCGCCAAGCTCTTGGCACGGCTGGCC

CATCACCTGCCCAACAAACAAACCCCGGCGCGGCACGCGCCAAGGAACTTTTGCATCATA

AGAAGGATAAGGCTCTCGGCTGTGCCGGAAATCGGACAACACGGTCGGGGGCTTCCTTCT

ATTCATAACAATAACGACTCTCGGCAACGGATATCTAGGCTCTTGCATCGATGAAGAACG

TAGCGAAATGCGATACTTGGTGTGAATTGCAGAATCCCGTGAACCATCGAGTCTTTGAAC

GCAAGTTGCGCCCGAAGCCTTCTGGCCGAGGGCACGCCTGCCTGGGTGTCACACATCGTC

GCCCCCCAAAATCCCGATATCTCGCCAGAGACAATCGGGAAGAGGATGGGCGGAAAATGG

TCTCCCGTGCGCTCCCGTTCGCGGTTGGCCCAAAAATGAGTTCCTGGCAAAGCAAAGCCA

CGACCAGCGGTGGTTGTAAGACCCTCGGTACAAGTCGTGAGCCTTGCCTTGCTCGTAGGG

ACATGTTGACCCTGAACGTGATCGATTAACATCGAACACTCACAAA

>H._delphicum

TCGAAACCTCCAAATGACCCGCGAACTAGTTATCCACAAGTCGGGGGTGTCGTGGGCTTC

TGTCCCGGCGCCCCCGTGCCGGTGGTGGCCAGGCGCGCCAAGCTCTTGGCACGGCTGGCC

CATCACCTGCCCAACAAACAAACCCCGGCGCGGCACGCGCCAAGGAACTTTTGCATCATA

AGAAGTGTAAGGCTCTCGGCTGTGCCGGAAATCGGACAACACGGTCGGGGGCTTCCTTCT

GTTCATAACAATAATGACTCTCGGCAACGGATATCTAGGCTCTTGCATCGATGAAGAACG

TAGCGAAATGCGATACTTGGTGTGAATTGCAGAATCCCGTGAACCATCGAGTCTTTGAAC

GCAAGTTGCGCCCGAAGCCTTCTGGCCGAGGGCACGCCTGCCTGGGTGTCACACATCGTC

GCCCCCCAAAATTCCGATATCTCGCCAGAGACAATCGGGAAGAGGATGGGCGGAAAATGG

TCTCCCGTGCACTCCCGTTCGCGGTTGGCCCAAAAATGAGTTCCTGGCAAAGCAAAGCCA

CGACCAGCGGTGGTTGTAAGACCCTCGGTACAAGTCGTGAGCCTTGCATTGCTCGTAGGG

ACATGTTGACCCTGAACGTGATCGAGTAACACCGAACACTCACAAA

>H._tetrapterum

TCGAAACCTCCAAATGACCCGCGAACTAGTTATCCACAAGTCGGGGGTGTCGTGGGCTTC

TGTCCCGGCGCCCCCGTGCCGGTGGTGGCCAGGCGCGCCAAGCTTTTGGCACGGCTGGCC

CATCACCTGCCCAACAAACAAACCCCGGCGCGGCACGCGCCAAGGAACTTTTGCATCATA

AGAAGTGTAAGGCTCCCGGCTGTGCCGGAAATCGGACAACACGGTCGGGGGCTTCCTTCT

GTTCATAACAATAACGACTCTCGGCAACGGATATCTAGGCTCTTGCATCGATGAAGAACG

TAGCGAAATGCGATACTTGGTGTGAATTGCAGAATCCCGTGAACCATCGAGTCTTTGAAC

GCAAGTTGCGCCCGAAGCCTTCTGGCCGAGGGCACGCCTGCCTGGGTGTCACACATCGTC

GCCCCCCAAAATCCCGGTATCTCGCAAGAGACAATCGGGAATAGGATGGGCGGAAAATGG

TCTCCCGTGCGCTCCCGTTCGCGGTTGGCCCAAAAATGAGTTCTTGGCAAAGCAAAGCCA

CGACCAGCGGTGGTTGTAAGACCCTCGGTACAAGTCGTGAGCCTTGCATTGCTCGTAGGG

ACATGTTGACCCTGAACGTGATCGAGTAACATCGGTACACTCACAAA

>H._undulatum

TCGAAACCTCCAAATGACCCGCGAACTAGTTATCCACAAGTCGGGGGTGTCGTGGGCTTC

TGTCCCGGCGCCCCCGTGCCGGTGGTGGCCAGGCGCGCCAAGCTTTTGGCACGGCTGGCC

CATCACCTGCCCAACAAACAAACCCCGGCGCGGCACGCGCCAAGGAACTTTTGCATCATA

AGAAGTGTAAGGCTCCCGGCTGTGCCGGAAATCGGACAACACGGTCGGGGGCTTCCTTCT

GTTCATAACAATAACGACTCTCGGCAACGGATATCTAGGCTCTTGCATCGATGAAGAACG

TAGCGAAATGCGATACTTGGTGTGAATTGCAGAATCCCGTGAACCATCGAGTCTTTGAAC

GCAAGTTGCGCCCGAAGCCTTCTGGCCGAGGGCACGCCTGCCTGGGTGTCACACATCGTC

GCCCCCCAAAATCCCGATATCTCGCAAGAGACAATCGGGAATAGGATGGGCGGAAAATGG

TCTCCCGTGCGCTCCCGTTCGCGGTTGGCCCAAAAATGAGTTCCTGGCAAAGCAAAGCCA

CGACCAGCGGTGGTTGTAAGACCCTCGGTACAAGTCGTGAGCCTTGCATTGCGCGTAGGG

ACATGTTGACCCTGAACGTGATCGAGTAACATCGGTACACTCACAAA

>H._attenuatum

TCGAAACCTCCAAATGACCCGCGAACTAGTTATCCCTGCGAACCTGCGGGGGTGTTGTGG

GCTTCTGTCCCGGCGCCCCCGTGCCGGTGGTGGCCAGGCACGCCAAGCTCTTGGCACGGC

TGGCCCATCACCTGCCCAACAAACAAACCCCGGCGCGGCACGCGCCAAGGAACTTTTGCA

TCATAAGAAGTGTAAGGCTCCCGGCTGTGCCGGAAATCGGACAACACGGTCGGGGGCTTC

CTTCTGTTCATAACAATAACGACTCTCGGCAACGGATATCTAGGCTCTTGCATCGATGAA

GAACGTAGCGAAATGCGATACTTGGTGTGAATTGCAGAATCCCGTGAACCATCGAGTCTT

TGAACGCAAGTTGCGCCCGAAGCCTTCTGGCCGAGGGCACGCCTGCCTGGGTGTCACACA

TCGTCGCCCCCCAAAATCCCGATATCTCGCCAGAGACAATCGGGAAGAGGATGGGCGGAA

AATGGTCTCCCGTGCGCTCCCGTTCGCGGTTGGCCCAAAAATGAGTTCCTGGCAAAGCAA

AGCCACGACCAGCGGTGGTTGTAAGACCCTCGGTACAAGTCGTGAGCCTTGCATTGCTCG

TAGGGACATGTTGACCCTGAACGTGATCGAGTAACATCGAACACTCACAAA

>H._olympicum

TCGAAACCTCCAAATGACCCGCGAACTAGTTATCCACAAGTCGGGGGTGTCGTGGGCTTC

TGTCCCGGCGCCCCCGTGCCGGTGGTGAYCAGTCGTGCCAAGCTCTTGGCATGGCTGGCC

CATCACCTGCCCAAAAAACAAACCCCGGCGCGGCACGCGCCAAGGAACTTTTGCATCATA

AGAAGGATCACGCTCCCCGGCTGTGCCGGAAATCGGACAACACGGTCGGGGGCTTCCTTC

TGTTYATAACAATAACGACTCTCGGCAACGGATATCTAGGCTCTTGCATCGATGAAGAAC

GTAGCGAAATGCGATACTTGGTGTGAATTGCAGAATCCCGTGAACCATCGAGTCTTTGAA

CGCAAGTTGCGCCCGAAGCCTTCTGGCCGAGGGCACGCCTGCCTGGGTGTCACACATCGT

CGCCCCCCAAAATCCCGATATCTCGCAAGAGACAATCGGGAAGAGGATGGGCGGAAAATG

GTCTCCCGTGCGCTCCCGTTCGCGGTTGGCCCAAAAATGAGTTCCTGGCAAAGCAAAGCC

ACGACCAGCGGTGGTTGTAAGACCCTCGGTACAAGTCGTGAGCCTTGCATTGCTTGTAGG

GACATATTGACCCTGAACGTGATCGAGTAACATCGAACACTCACAAA

>H._hirsutum

TCGAAACCTCCAAATGACCCGCGAACCAGTTATCCACAAGTGGGGGTGTCGGGCTTTGTC

CCGGCGCCCCCGTTCGGGTGGTGGTCAGGCGCGCCAAGCTCTTGGCACGGCTGTTCCATC

ACCTGCCCAACAAACAAACCCCGGCGCGGCACGCGCCAAGGAACTTTTGCAATATAAGAA

GGATCACGCTCCCGGCCGTGCCGGAAATCGGACAACGCGGTCGGGGGCTTCCTTCTATTC

ATAATAATAACGACTCTCGGCAACGGATATCTAGGCTCTTGCATCGATGAAGAACGTAGC

GAAATGCGATACTTGGTGTGAATTGCAGAATCCCGTGAACCATCGAGTCTTTGAACGCAA

GTTGCGCCCGAAGCCTTCTGGCCGAGGGCACGCCTGCCTGGGTGTCACACATCGTCGCCC

CCCAAAATCCGATCTCTCGCAAGGGACGATCGGGAAGAGGATGGGCGGAAAATGGTCTCC

CGTGCGCTCCCGTTCGCGGTTGGCCCAAAAATGAGTTCCTGGCAAAGCAAAGCCACGACC

AGCGGTGGTTGTAAGACCCTCGGTACAAGTCGTGAGCCTTGCGTTGCGATTACGGACATG

TTGACCCTGAACGTGATCGAGTAACTTCGAACGCTCACAAA

>H._acmosepalum

TCGAAACCTCCAAATGACCCGCGAACCAGTTATCCACAAGTCGGGGGTGTCGGGCTGAAC

CCCGTAACCCCCGTGCGCCGGTGGCGGTCAGGCGTGCCAAGCTCTTGGCACGGTTGGCCC

GTCACCTGCCCAACAAACCAACCCCGGCGCGGCACGCGCCAAGGAACTTTGCATCATGAG

AAGGACAATGCCCCCGTCCGTGCCGGAAATCGGATAACGCGGCCGGTGGCTTTCCTTCGG

TTCATAACTAAAACGACTCTCGGCAACGGATATCTAGGCTCTTGCATCGATGAAGAACGT

AGCGAAATGCGATACTTGGTGTGAATTGCAGAATCCCGTGAACCATCGAGTCTTTGAACG

CAAGTTGCGCCCGAAGCCTTCTGGCCGAGGGCACGTCTGCCTGGGTGTCACACATCGTCG

CCCCCAAAACCAATGCCTCTTTCGAGTTCATTGGGTACAGGATGGGCGGATAATGGTCTC

CCGTGCGCACCCGTTCGCGGTTGGCCCAAAACTTTGTTCCTGGCGATCGCAAGCCATGAC

CAGCGGTGGTTGTAAGACCCTCGGTCATAGTCGTGAGCTTGCACGTCGGGACTCATCGAC

CCTGAACGTGATTGAGAAACCTCGAACACTCACAAA

>H._patulum

TCGAAACCTCCAAATGACCCGCGAACCAGTTATCCACAAGTCGGGGGTGTCGGGCTGAAC

CCCGTAACCCCCGTGCGCCGGTGGCGGTCAGGCGTGCCAAGCTCTTGGCACGGTTGGCCC

GTCACCTGCCCAACAAACCAACCCCGGCGCGGCACGCGCCAAGGAACTTTGCATCATGAG

AAGGACAATGCCCCCGTCCGTGCCGGAAATCGGATAACACGGCCGGTGGCTTTCCTTCTG

TTCATAACTAAAACGACTCTCGGCAACGGATATCTAGGCTCTTGCATCGATGAAGAACGT

AGCGAAATGCGATACTTGGTGTGAATTGCAGAATCCCGTGAACCATCGAGTCTTTGAACG

CAAGTTGCGCCCGAAGCCTTCTGGCCGAGGGCACGTCTGCCTGGGTGTCACACATCGTCG

CCCCCAAAACCAATGCCTCACTCGAGTTCATTGGGTACAGGATGGGCGGATAATGGTCTC

CCGTGCGCACCCGTTCGCGGTTGGCCCAAAACTTTGTTCCTGGCGATCGCAAGCCATGAC

CAGCGGTGGTTGTAAGACCCTCGGTCATAGTCGTGAGCTTGCACGTCGGGACATATCGAC

CCTGAACGTGATCGAGAAACCTCGAACACTCACAAA

>H._kouytchense

TCGAAACCTCCAAATGACCCGCGAACCAGTTATCCACAAGTCGGGGGTGTCGGGCTGAAC

CCCGTAACCCCCGTGCGCCGGTGGCGGTCAGGCGTGCCAAGCTCTTGGCACGGTTGGCCC

GTCACCTGCCCAACAAACCAACCCCGGCGCGGCACGCGCCAAGGAACTTTGCATCATGAG

AAGGACAATGCCCCCGTCCGTGCCGGAAATCGGATAACGCGGCCGGTGGCTTTCCTTCTG

TTCATAACTAAAACGACTCTCGGCAACGGATATCTAGGCTCTTGCATCGATGAAGAACGT

AGCGAAATGCGATACTTGGTGTGAATTGCAGAATCCCGTGAACCATCGAGTCTTTGAACG

CAAGTTGCGCCCGAAGCCTTCTGGCCGAGGGCACGTCTGCCTGGGTGTCACACATCGTCG

CCCCCAAAACCAATGCCTCACTCGAGTTCATTGGGTACAGGATGGGCGGATAATGGTCTC

CCGTGCGCACCCGTTCGCGGTTGGCCCAAAACTTTGTTCCTGGCGATCGCAAGCCATGAC

CAGCGGTGGTTGTAAGACCCTCGGTCATAGTCGTGAGCTTGCACGTCGGGACATATCGAC

CCTGAACGTGATCGAGAAACCTCGAACACTCACAAA

>H._calycinum

TCGAAACCTCCAAATGACCCGCGAACGAGTTATCCACAAGTCGGGGGTGTCGGGCTAAAC

CCCGTAACCCCCGTGCGCCGGTGGCGATCAGGCGTGCCAAGCTCTTGGCACGGTTGGCCC

GTCACCTGCCCAACAAACCAACCCCGGCGCGGCACGCGCCAAGGAACTTTGCATCGTGAG

AAGGATAACGCTCCGTCCGTGCCGGAAATCGGATAACACGGCCGGTGGCTTTCCTTCTGT

TCATAACCAAAACGACTCTCGGCAACGGATATCTAGGCTCTTGCATCGATGAAGAACGTA

GCGAAATGCGATACTTGGTGTGAATTGCAGAATCCCGTGAACCATCGAGTCTTTGAACGC

AAGTTGCGCCCGAAGCCTTCTGGCCGAGGGCACGTCTGCCTGGGTGTCACACATCGTCGC

CCCCAAACCCAATGCCTCACTCGAGTTTATTGGGTATAGGATGGGCGGATAATGGTCTCC

CGTGCGCACCCGTTCGCGGTTGGCCCAAAACTTTGTTCCTGGCGATCGCAAGCCATGACC

AGCGGTGGTTGTAAGACCCTCGGTTATAGTCGTGAGCTTGCACGTCGGGACGTATCGACC

CTGAACGTGCTCGAGAAACCTCGAACACTCACAAA

>H._athoum

TCGAAACCTCCAAATGACCCGCGAACTAGTTATCCACAAGTCGGGGGTGTCGTGGGCTTC

TGTCCCGGCGCCCCCGTGCCGGTGGTGGCCAGGCGCGCCAAGCTCTTGGGACGGCTGGCC

CATCACCTGCCCAACAAACAAACCCCGGCGCGGCACGCGCCAAGGAACTTTTGCATCATA

AGAAGTGTAAGGCTCTCGGCTGTGCCGGAAATCGGACAACACGGTCGGGGGCTTCCTTCT

GTTCATAACAATAACGACTCTCGGCAACGGATATCTAGGCTCTTGCATCGATGAAGAACG

TAGCGAAATGCGATACTTGGTGTGAATTGCAGAATCCCGTGAACCATCGAGTCTTTGAAC

GCAAGTTGCGCCCGAAGCCTTCTGGCCGAGGGCACGCCTGCCTGGGTGTCACACATCGTT

GCCCCCCGAAATTCCGATATCTCGCCAGAGACAATCGGGAAGAGGATGGGCGGAAAATGG

TCTCCCGTGCGCTCCCGTTCGCGGTTGGCCCAAAAATGAGTTCCTGGCAAAGCAAAGCCA

CGACTAGCGGTGGTTGTAAGACCCTCGGTACAAGTCGTGAGCCTTGCATTGCTCGTAGGG

ACATGTTGACCCTGAACGTGATCGAGTAACACCGAACACTCACAAA

>H._androsaemum

TCGAAACCTCCAAACGACCCGCGAACTAGTTATCCACAAGTCGGGGGTGTCGGGTTTATC

CCCGTAACCCCCGTGCGCCGGTGGTGGCCATGCGAGCCGATCTCTCGGCACGGTTGGCCC

ACCACCTGCCCAACAAACCAACCCCGGCGCGGCACGCGCCAAGGAACCTTGCATCATAAG

AAGGATCACGCTCCCGTCCGTGCCGGAAATCGGATAACGCGGTCGGCGGCTGTCCTCCTG

TTCATAACAAAAACGACTCTCGGCAACGGATATCTTGGCTCTTGCATCGATGAAGAACGT

AGCGAAATGTGATACTTGGTGTGAATTGCAGAATCCCGTGAACCATCGAGTCTTTGAACG

CAAGTTGCGCCCGAAGCCTTCTGGCCGAGGGCACGTCTGCCTGGGTGTCACACATCGTCG

CCCCAAACCCAACACCTCGCCAGAGGAGCTTGGGAAGAGGATGGGGCGGATAATGGTCTC

CCGTGCGCACCCGTTCGCGGTTGGCCCAAAAATCAGTTCCTGGCGATCGCAAGCCACGAC

CAGCGGTGGTTGTAAGACCCTCGATTTCAAGTCGTGAGCACCTGCCTCGCATGTCGGGAC

AATTTGACCCCGTACGTGCTCGAATAACTTCGAACACTCACAAA

>H._ascyron

TCGAAGCCTCCAAACGACCCGGGAACCGGTTATCCACATGTCGGGGGCGTCGGGCTGAAC

CCCGCGACCCCCGTGCGCGGGCGGCGGTCGGGCGTGCCAAGATCTTGGCACGGCCGGCCC

GTCACCCGCCCAACAAACCAACCCCGGCGCGGCACGCGCCAAGGAACTTTGCATAACGAG

GAGGACGACGCTCCCGGTCGTGCCGGAAATCGGACTACACGGCCGGTGGCTCTCCTTCTG

TTCATAACCAAAACGACTCTCGGCAACGGATATCTAGGCTCTTGCATCGATGAAGAACGT

AGCGAAATGCGATACTTGGTGTGAATTGCAGAATCCCGTGAACCATCGAGTCTTTGAACG

CAAGTTGCGCCCGAAGCCTTCTGGCCGAGGGCACGTCTGCCTGGGTGTCACACAACGTCG

CCCCCAAACCCAACGACTCGCTCGAGTTCGCTGGGCACCCGAGGGGCGGATAATGGTCTC

CCGTGCGCTCCCGTTCGCGGTTGGCCCAAAACTTTGTTCCCGGCGATCGCAAGCCACGAC

AAGCGGTGGTTGTAAGACCCTCGGTAATCGTCGTGAGCCCGCGCGCCGGGACACATCGAC

CCCGAACGCGTTCCGGGGGAGACCCCGAACGCTCACAAA

>H._japonicum

TCGAAACTTGCAAAAGAAGACCCGTGAACTAGTTATCAACATGTGGGGGGAGTGTTGGGA

GATATCCCTTATAGCTCCTTCGTGCCAGTGGTGGGTAATCGTGTCGTTATACCGGCATGA

TTGACCAACATCTGGACAAGCTAACCAACCCCGGCGCGGCATGCGCCAAGGAACTTTGCA

ATAAATGTGGAAACGCTACCGGTCGTTCCGAAAATCGGATAAAGCGGCCGTGTTGTGGAC

CACTTCTTCATTACAAAACGACTCTCGGCAACGGATATCTAGGCTCTTGCATCGATGAAG

AACGTAGCGAAATGCGATACTTGGTGTGAATTGCAGAATCCCGTGAACCATCGAGTCTTT

GAACGCAAGTTGCGCCCGAAGCCTCTTGGCCAAGGGCACGTCTGCCTGGGTGTCACACAT

CGTCCCCACCGAAACAAATGCTCCTTGTAGGTGTTTGGTAAGAGGATGGGCGGATAATGG

TCTCCCGTGCGCGGTTGCTCGCGGTTGGCCGAAAAATGAGTTCCTGGTGATAGCAAGCCA

TGACCAGCGGTGGTTGAAAGACCCTCGATGGGTGTCGTGAGAACTTGCATCGCATGTTGG

GAGATTCTGACCTTGATCGTGTTTGAGCATATATCAAACACTCATGAA

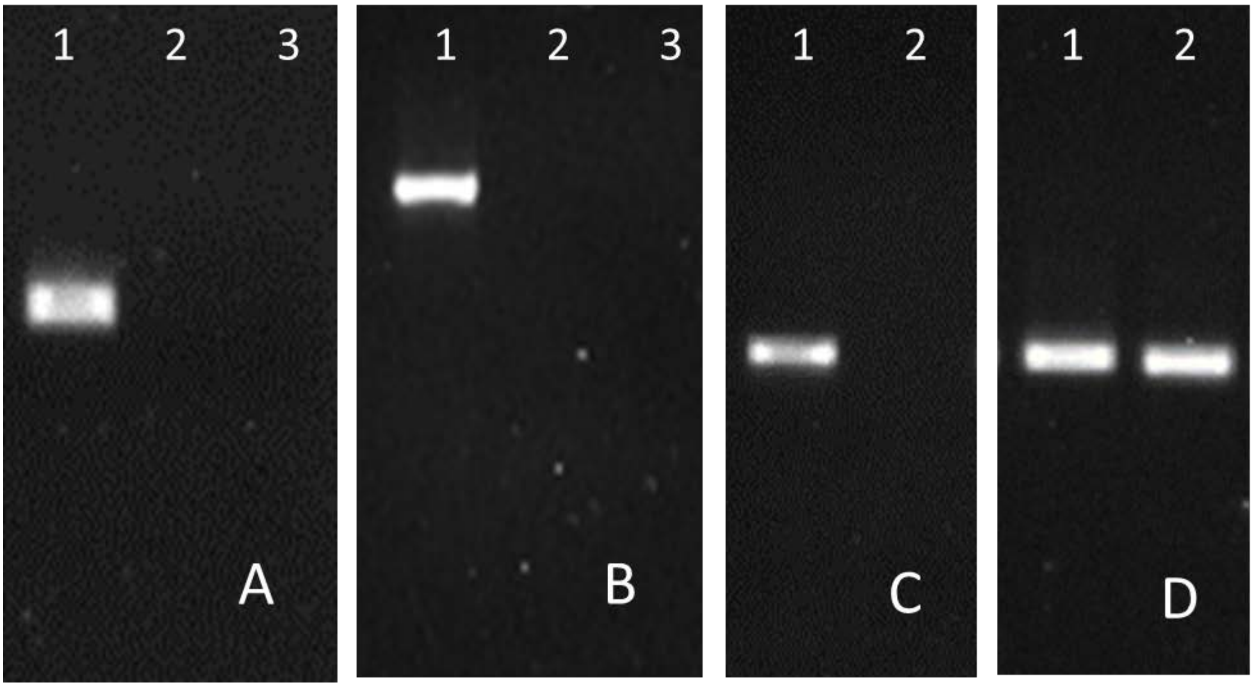

Supplementary Information, Figure S1: Agarose gel electrophoresis of PCRs showing specificity of the new generation primers. Gel lanes: 1. genomic DNA from 1456, *H. perforatum* ; 2. genomic DNA from 1479, *H. patulum*; 3. Negative (no template) control. Primer pairs: A, 460-650; B, FO2-650; C, FO2-460; D, HypGF-HypGR

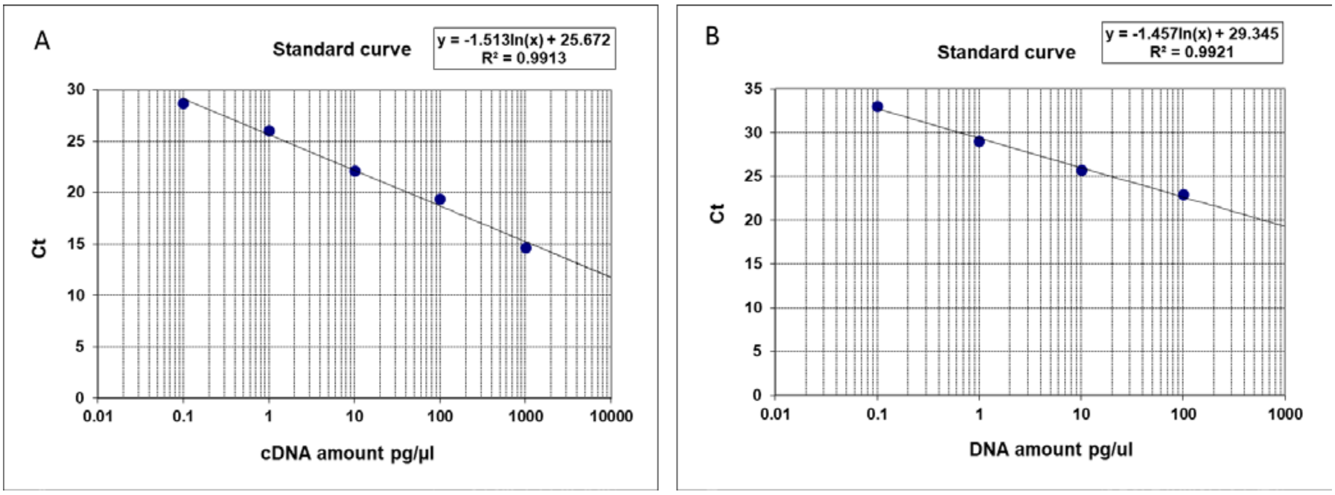

Supplementary Information, Figure S2: Quantitative Real-time PCR standard curve *Hypericum perforatum* serial dilutions with the A) generic HypG F/R primers and the B) specific 460F-650R primers.

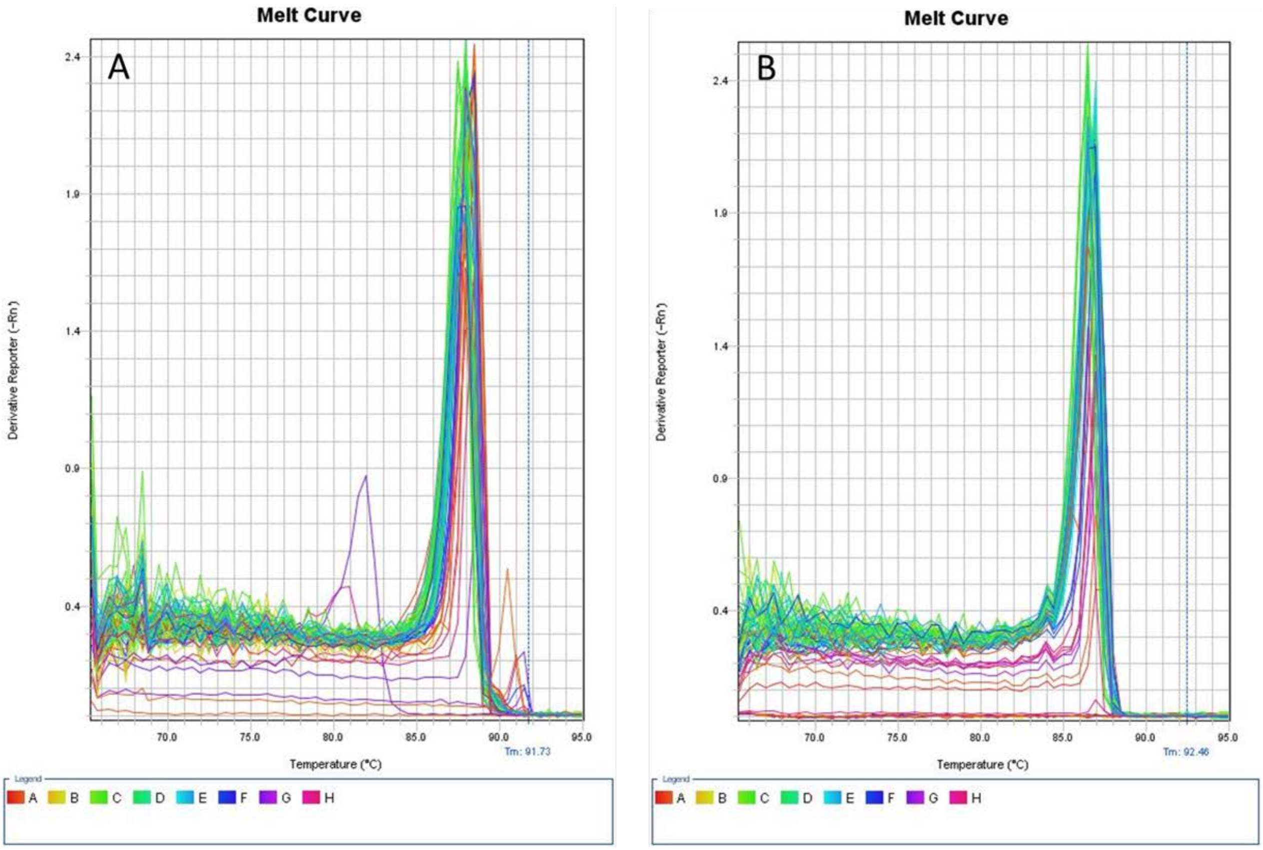

Supplementary Information, Figure S3: Quantitative Real-time PCR Melting curve generated with the A) generic HypG F/R primers and the B) specific 460F-650R primers
